# Periplasmic carbonic anhydrase CAH1 contributes to high inorganic carbon affinity in *Chlamydomonas reinhardtii*

**DOI:** 10.1093/plphys/kiae463

**Published:** 2024-08-30

**Authors:** Daisuke Shimamura, Tomoaki Ikeuchi, Ami Matsuda, Yoshinori Tsuji, Hideya Fukuzawa, Keiichi Mochida, Takashi Yamano

**Affiliations:** Graduate School of Biostudies, Kyoto University, Kyoto 606-8502, Japan; RIKEN Center for Sustainable Resource Science, Yokohama 230-0045, Japan; Graduate School of Biostudies, Kyoto University, Kyoto 606-8502, Japan; Graduate School of Biostudies, Kyoto University, Kyoto 606-8502, Japan; Graduate School of Biostudies, Kyoto University, Kyoto 606-8502, Japan; Department of Bioscience, School of Biological and Environmental Sciences, Kwansei Gakuin University, Hyogo 669-1330, Japan; Graduate School of Biostudies, Kyoto University, Kyoto 606-8502, Japan; RIKEN Center for Sustainable Resource Science, Yokohama 230-0045, Japan; Graduate School of Biostudies, Kyoto University, Kyoto 606-8502, Japan; Center for Living Systems Information Science (CeLiSIS), Kyoto University, Kyoto 606-8501, Japan

## Abstract

Carbonic anhydrase (CA), an enzyme conserved across species, is pivotal in the interconversion of inorganic carbon (Ci; CO_2_, and HCO_3_^−^). Compared to the well-studied intracellular CA, the specific role of extracellular CA in photosynthetic organisms is still not well understood. In the green alga *Chlamydomonas* (*Chlamydomonas reinhardtii*), carbonic anhydrase 1 (CAH1), located at the periplasmic space, is strongly induced under CO_2_-limiting conditions by the Myb transcription factor LCR1. While the *lcr1* mutant shows decreased Ci-affinity, the detailed mechanisms behind this phenomenon are yet to be elucidated. In this study, we aimed to unravel the LCR1-dependent genes essential for maintaining high Ci-affinity. To achieve this, we identified a total of 12 LCR1-dependent inducible genes under CO_2_-limiting conditions, focusing specifically on the most prominent ones—*CAH1*, *LCI1*, *LCI6*, and *Cre10.g426800*. We then created mutants of these genes using the CRISPR–Cas9 system, all from the same parental strain, and compared their Ci-affinity. Contrary to earlier findings that reported no reduction in Ci-affinity in the *cah1* mutant, our *cah1-1* mutant exhibited a decrease in Ci-affinity under high HCO_3_^−^/CO_2_-ratio conditions. Additionally, when we treated wild-type cells with a CA inhibitor with low membrane permeability, a similar reduction in Ci-affinity was observed. Moreover, the addition of exogenous CA to the *cah1* mutant rescued the decreased Ci-affinity. These results, highlighting the crucial function of the periplasmic CAH1 in maintaining high Ci-affinity in *Chlamydomonas* cells, provide insights into the functions of periplasmic CA in algal carbon assimilation.

## Teaser

A periplasmic carbonic anhydrase in *Chlamydomonas reinhardtii* plays a crucial role in maintaining a high affinity for inorganic carbon, particularly under CO_2_-limiting conditions.

## Introduction

Carbonic anhydrase (CA; EC 4.2.1.1) is a metalloenzyme that catalyzes the interconversion between CO_2_ and HCO_3_^−^. CA is among the enzymes that display the highest turnover rates (Chegwidden and Carter 2000), thereby fulfilling biological demands in diverse physiological processes such as pH homeostasis, inorganic carbon (Ci; CO_2_ and HCO_3_^−^) transport, and Ci assimilation. CA is classified into eight subclasses based on the primary structure (Aspatwar et al. 2022).

In land plants, CA is hypothesized to play a crucial role in carbon assimilation, although its function remains controversial. Historically, it has been posited that in C3 plants, abundant CA in the chloroplast stroma aids CO_2_ fixation by facilitating its diffusion (Jacobson et al. 1975). However, this understanding has been challenged by recent studies. For instance, Hines et al. (2021) found that the complete loss of stromal CA does not significantly alter carbon assimilation compared to wild-type (WT) plants. In contrast, CAs' role in aquatic organisms, such as microalgae and cyanobacteria, is more clearly defined within the operation of the CO_2_-concentrating mechanism (CCM) (Fukuzawa et al. 1992; Badger 2003). Notably, Rubisco, a key enzyme in photosynthetic CO_2_ fixation, exhibits a lower affinity for CO_2_ in microalgae and cyanobacteria than in its terrestrial counterparts (Jordan and Ogren 1981). In these aquatic organisms, the CCM helps overcome the disadvantage of Rubisco's lower CO_2_ affinity by actively transporting HCO_3_^−^ into the chloroplast stroma through membrane transporters and channels. Once in proximity to Rubisco, HCO_3_^−^ is converted to CO_2_ by intracellular CA, effectively concentrating CO_2_ (Raven et al. 2011).

In *Chlamydomonas* (*Chlamydomonas reinhardtii*), a freshwater green alga, CAs are crucial for driving CCM, and the compartmentalized CAs are integral to supplying CO_2_ specifically to the pyrenoid, where Rubisco is densely packed in the chloroplast (Moroney et al. 2011). Among them, carbonic anhydrase 3 (CAH3), an α-type CA, plays a unique role. It is localized in the lumen of the pyrenoid-invading thylakoid membrane, also known as the pyrenoid tubule (Sinetova et al. 2012). Here, CAH3 facilitates the conversion of HCO_3_^−^ to CO_2_, a process enhanced by the lumen's acidic pH. CAH3-deficient mutant exhibits decreased Ci affinity with higher accumulation of internal Ci relative to WT cells, highlighting its role in the generation of CO_2_ from the stromal Ci pool (Funke et al. 1997; Karlsson et al. 1998). Additionally, the low-CO_2_ inducible protein B/C (LCIB/C) hexamer, a θ-type CA positioned around the pyrenoid, serves to reconvert CO_2_ leaking from the pyrenoid into HCO_3_^−^, maintaining optimal Ci concentration for photosynthesis (Wang and Spalding 2006; Yamano et al. 2010; Kasili et al. 2023). In addition to LCIB/LCIC and CAH3, *Chlamydomonas* has α-type CAs (CAH1 and CAH2), β-type CAs (CAH4 to CAH9), and γ-type CAs (CAG1 to CAG3), but their roles in CCM remain unresolved (Moroney et al. 2011).

CAH1, an α-type CA localized at the periplasmic space, was the first CA to be identified in *Chlamydomonas* (Coleman et al. 1984), but its importance in the CCM remains controversial. CAH1 is induced upon CO_2_-limitation, and its induction is dependent on a Myb transcription factor LCR1, whose expression is regulated by CCM1/CIA5, a master regulator of CCM (Fukuzawa et al. 1990, 2001; Xiang et al. 2001; Yoshioka et al. 2004). In addition to the abundant accumulation of CAH1 under CO_2_-limiting conditions, inhibition of periplasmic CA by weakly permeable CA inhibitors, such as acetazolamide (AZA), has been shown to decrease Ci-affinity (Moroney et al. 1985). While other CA isoforms, including the α-type CAH2 and the β-type CAH8, are also present in the periplasmic space, their expression levels under CO_2_-limiting conditions are lower compared to that of *CAH1* (Moroney et al. 2011). These observations have led to the establishment of a well-known model in which periplasmic CA, particularly CAH1, facilitates diffusive CO_2_ entry by maintaining a CO_2_ gradient across the plasma membrane. This is achieved through the rapid equilibration of CO_2_ with bulk HCO_3_^−^ at the cell surface. Because periplasmic CA activity was detected in diverse algae (Nimer et al. 1999; Elzenga et al. 2000; Tsuji et al. 2017, 2021), and its inhibition by AZA caused the decline of Ci-affinity, periplasmic CA-mediated CO_2_ uptake has been a widespread hypothesis. Conversely, it has also been suggested that the effects of AZA on photosynthetic kinetics may be due to the inhibition of intracellular CAs rather than extracellular ones (Williams and Turpin 1987). Moreover, a *Chlamydomonas* mutant lacking CAH1 showed no difference in growth or Ci-affinity difference under low-CO_2_ conditions (Van and Spalding 1999), challenging the hypothesis that periplasmic CA facilitates CO_2_ acquisition from bulk HCO_3_^−^. Another hypothesis based on the mathematical modeling is that periplasmic CA recaptures leaked CO_2_ through hydration reaction (Fridlyand 1997). Thus, while massive effort has been spent to elucidate the function of periplasmic CA, conclusive evidence to support either hypothesis has not been presented yet.

We previously demonstrated through macroarray analysis, which is limited to a specific number of genes, that the *lcr1* mutant was unable to induce at least three low-CO_2_ (LC) inducible genes, namely *CAH1*, *LCI1*, and *LCI6* (Yoshioka et al. 2004). Notably, LCI1, localized at the plasma membrane, is hypothesized to function as a CO_2_ channel due to its structural characteristics (Ohnishi et al. 2010; Kono et al. 2020). In addition, LCI1 interacts with high-light activated protein 3 (HLA3), an HCO_3_^−^ transporter on the plasma membrane (Yamano et al. 2015; Mackinder et al. 2017). Although the *lcr1* mutant shows a decrease of Ci-affinity under CO_2_-limiting conditions, the major contributor to this phenotype has not been determined yet. Among the three candidates, independent disruption of *CAH1* and *LCI1* do not show decreased Ci-affinities (Van and Spalding 1999; Kono and Spalding 2020), suggesting that cooperative functions of these three components or contribution of other unidentified factors for high-affinity photosynthesis for Ci. In this study, to gain further insight into *LCR1*-dependent CCM factors, we generated a *lcr1* mutant and identified LCR1-dependent genes by RNA-seq analysis. Furthermore, by generating mutant strains of *LCR1*-dependent genes using the CRISPR–Cas9 method, we found that loss of CAH1 causes a decrease in Ci-affinity.

## Results

### Identification of LCR1-dependent inducible genes under CO_2_-limiting conditions

**Figure 1.**
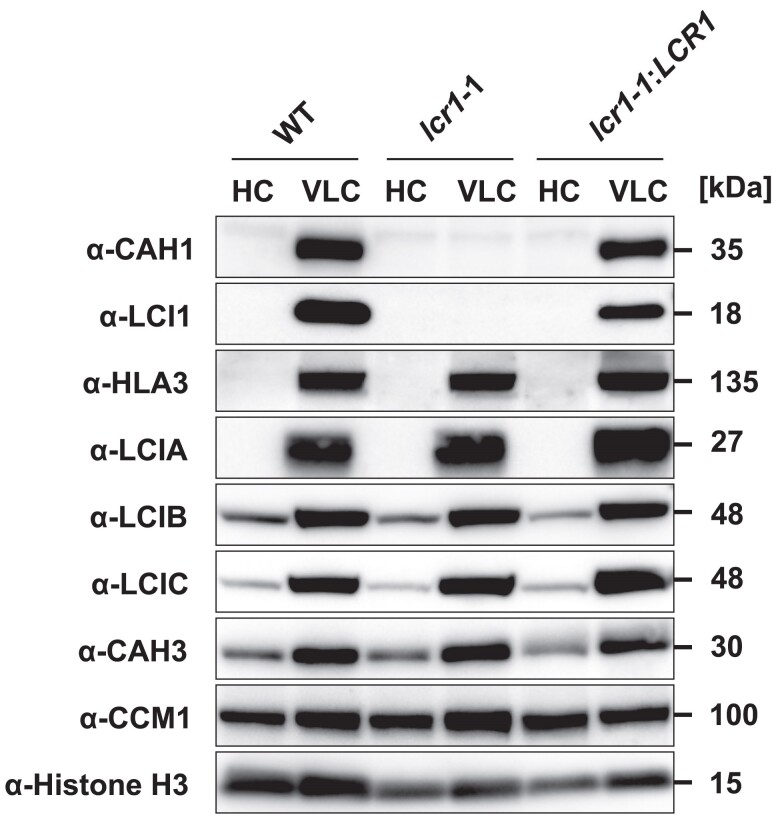
Accumulation of CCM-related proteins in the *lcr1* mutant. Cells were first grown under 5% (v/v) CO_2_ condition for 24 h and shifted to 5% (v/v) CO_2_ (HC) or 0.04% (v/v) (VLC) CO_2_ conditions for 12 h at pH 7.0. Histone H3 was used as a loading control. WT, wild type.

In our previous study (Yoshioka et al. 2004), we utilized the *lcr1* insertion mutant derived from the parental strain Q304P3, where *CAH1*-promoter activity was monitored by arylsulfatase (Ars) enzyme activity (Kucho et al. 1999). However, due to the absence of a cell wall, this strain was unsuitable for physiological analysis. To address this, we generated a *lcr1* mutant, named *lcr1*-1 in this study, derived from the WT strain C9 with a cell wall. To create the *lcr1*-1 mutant, we inserted the *AphVII* gene cassette, conferring hygromycin resistance, into the first exon of *LCR1* using the CRISPR–Cas9 system (Supplementary Fig. S1A and B). The *lcr1*-1:*LCR1* complemented strain was also created by reintroducing the *LCR1* gene fragment, including its putative promoter, 5′-UTR, and 3′-UTR, into *lcr1*-1. In the *lcr1*-1:*LCR1*, the reduced accumulation levels of CAH1 and LCI1 observed in the *lcr1*-1 were rescued (Fig. 1). On the other hand, the accumulation levels of HLA3, LCIA, LCIB, LCIC, CAH3, and CCM1 did not change among the strains, consistent with previous findings that LCR1 specifically regulates *CAH1* and *LCI1* among CCM-related genes (Yoshioka et al. 2004).

Given the limitations of macroarray analysis in previous studies for quantifying all gene expression levels (Yoshioka et al. 2004), we explored whether LCR1 influences genes beyond *CAH1*, *LCI1*, and *LCI6* under CO_2_-limiting conditions using RNA-seq analysis. We cultured WT, *lcr1*-1, and *lcr1*-1:*LCR1* cells under 5% CO_2_ (high-CO_2_; HC) or 0.04% CO_2_ aerated (very-low CO_2_; VLC) conditions in MOPS-P liquid medium at pH 7.0 and quantified their transcriptome profiles. In WT cells, the expression levels of 1,647 genes were significantly increased at either 0.3 or 2 h after switching to VLC conditions compared to HC conditions [(FDR) < 0.01 and log_2_FC < −1] (Supplementary Data Set 1). Among them, under VLC conditions, the expression levels of 12 genes, including *LCR1*, *CAH1*, *LCI1*, and *LCI6*, were significantly decreased in *lcr1*-1 and recovered in *lcr1*-1:*LCR1* (Table 1). Among these genes, *Cre10.g426800*, encoding a protein of unknown function with a transferase domain, was particularly notable as its expression level decreased more than 4-fold by *LCR1* mutation both 0.3 and 2 h after VLC induction (Table 1). This led us to focus our subsequent analysis on *CAH1*, *LCI1*, *LCI6*, and *Cre10.g426800*.

**Table 1.** Genes downregulated in the *lcr1* mutant under VLC conditions

Gene ID	Gene name	Description	VLC-0.3 h				VLC-2.0 h			
			*lcr1*-1/WT		*lcr1*-1/*lcr1*-1:*LCR1*		*lcr1*-1/WT		*lcr1*-1/*lcr1*-1:*LCR1*	
			log_2_FC	FDR	log_2_FC	FDR	log_2_FC	FDR	log_2_FC	FDR
*Cre02.g095065*			0.98	7.1.E–02	−0.54	7.8.E–01	−1.20	6.1.E–03	−1.78	1.9.E–04
*Cre02.g095067*			0.47	2.5.E–01	−0.52	7.1.E–01	−1.26	4.3.E–04	−1.70	3.6.E–05
*Cre03.g162800*	*LCI1*	Low-CO_2_-inducible membrane protein	−0.30	6.4.E–01	−0.80	7.0.E–01	−1.98	5.2.E–04	−2.03	3.1.E–03
*Cre04.g223100*	*CAH1*	Carbonic anhydrase	−3.49	5.9.E–27	−2.24	1.9.E–10	−2.86	3.0.E–19	−2.30	3.8.E–11
*Cre06.g278137*			−1.90	2.4.E–06	−0.05	9.9.E–01	−3.41	8.9.E–21	−1.85	1.0.E-04
*Cre08.g364050*			−0.10	7.6.E–01	−1.01	1.6.E–02	−1.04	9.2.E–05	−1.75	2.8.E–10
*Cre08.g381450*	*OPR35*	OctotricoPeptide Repeat Protein	−1.71	8.7.E–10	−1.23	2.6.E–03	−2.62	1.3.E–21	−1.47	8.5.E–06
*Cre09.g399552*	*LCR1*	Myb-like transcription factor	−2.54	4.4.E–05	−1.74	1.9.E–01	−2.55	6.3.E-05	−2.20	4.3.E-03
*Cre10.g426800*			−2.37	2.9.E–20	−2.12	2.3.E–14	−2.79	1.6.E–27	−2.20	7.4.E-16
*Cre10.g448200*	*ARL9*	ARF-like GTPase	−1.84	1.3.E–09	−0.45	7.3.E–01	−2.19	1.3.E–13	−1.14	3.0.E–03
*Cre12.g553350*	*LCI6*	Low-CO_2_-inducible protein 6	0.69	5.9.E–02	−0.96	1.9.E–01	−1.64	9.5.E–07	−1.43	4.9.E–04
*Cre16.g684022*			−2.68	2.8.E–20	−1.86	6.4.E–08	−2.34	1.1.E–15	−1.20	1.6.E–03

Differentially expressed genes in *lcr1*-1 cells, with false discovery rate < 0.01 and log_2_FC < −1, in 0.04% CO_2_ aerated conditions for 0.3 or 2 h were indicated. WT, wild type; VLC, very low-CO_2_.

### Impact of CAH1 and LCI1 mutations on Ci-affinity in *Chlamydomonas* cells

To clarify the contribution of LCR1-dependent genes to CCM, we employed the CRISPR–Cas9 method to generate mutants of *CAH1*, *LCI1*, *LCI6*, and *Cre10.g426800*. First, we created insertional mutants of *LCI6* and *Cre10.g426800* and measured their photosynthetic O_2_-evolution rates. Three strains with an insertional mutation in the first exon of *LCI6* were isolated. Additionally, two strains of *Cre10.g426800* were isolated: one with a mutation in the first exon and the other in the second exon (Supplementary Fig. S2, A and B). To evaluate Ci-affinity in these mutants, we measured their O_2_-evolving activity. The selected pH conditions of 6.2, 7.0, and 7.8 represent a range that encompasses typical environmental variations, allowing us to assess the mutants' responses under diverse but relevant scenarios. In these mutants, the *K*_0.5_ (Ci) values, the Ci concentrations required for half-maximal O_2_-evolving rate, were measured at pH 7.8, where CCM phenotypes are most pronounced. The mutants showed no increase in *K*_0.5_ (Ci) compared to the WT, unlike in the *lcr1*-1 strain (Supplementary Table S1). Furthermore, complementation of *lcr1* with these genes rescued the Ci-affinity to WT levels. These results suggest that these two genes were not important for maintaining high Ci-affinity under CCM-inducing conditions.

**Figure 2.**
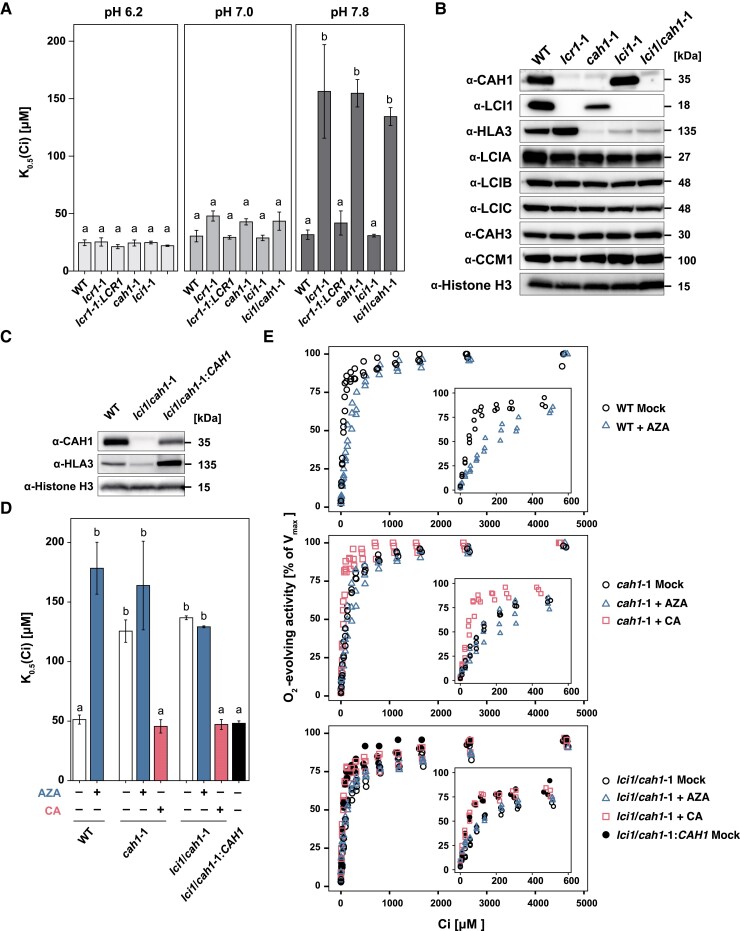
Physiological characteristics of *lcr1*, *cah1*, *lci1*, and *lci1*/*cah1* mutants. **A)***K*_0.5_ (Ci) values of *cah1*-1, *lci1*-1 and *lci1*/*cah1*-1 cells at pH 6.2, 7.0, and 7.8. Cells were grown in 0.04% (v/v) CO_2_ conditions for 12 h at pH 7.0. Data from all experiments show mean values ± standard error (Se) from three biological replicates. Statistical analysis was conducted using the Tukey–Kramer multiple comparison test, with different letters indicating significant differences (*P* < 0.05). **B)** Accumulation of CCM-related proteins in WT, *lcr1*-1, *cah1*-1, *lci1*-1 and *lci1*/*cah1*-1 cells grown under 0.04% (v/v) CO_2_ conditions for 12 h at pH 7.0. Histone H3 was used as a loading control. **C)** Accumulation of CAH1 and HLA3 in WT, *lci1*/*cah1*-1 and *lci1*/*cah1*-1:*CAH1* cells under 0.04% (v/v) CO_2_ condition at pH 7.0. Histone H3 was used as a loading control. **D)***K*_0.5_ (Ci) values in WT, *cah1*-1, *lci1*/*cah1*-1, and *lci1*/*cah1*-1:*CAH1* cells at pH 7.8. Cells were grown in 0.04% (v/v) CO_2_ conditions for 12 h at pH 7.0. Data from all experiments show mean values ± Se from three biological replicates. Values for cells treated with AZA or bovine CA are shown in blue and red bars, respectively. The Tukey–Kramer multiple comparison test was utilized for statistical analysis, with differing letters indicating statistically significant variations (*P* < 0.05). **E)** Oxygen-evolving activity of WT, *cah1*-1, *lci1*/*cah1*-1, and *lci1*/*cah1*-1:*CAH1* cells from three biological replicates treated with AZA or bovine CA in response to external dissolved Ci concentrations at pH 7.8 for the ranges of 0 to 5,000 *µ*m Ci and 0 to 600 *µ*m Ci (inset). Before measurements, cells were grown in the liquid culture aerated with 0.04% CO_2_ for 12 h. Values in each cell with AZA or bovine CA are shown as blue triangle and red square plots, respectively.

Next, we isolated mutants of *CAH1* and *LCI1*, designated *cah1*-1 and *lci1*-1, respectively, and also produced a double mutant (*lci1*/*cah1*-1) by disrupting the *CAH1* in the *lci1*-1 background (Supplementary Fig. S3, A and B). The *K*_0.5_ (Ci) values of the mutants were similar to those of the WT at pH 6.2 and 7.0, with significant differences emerging only at pH 7.8 (*P* < 0.05). At this pH, the *K*_0.5_ (Ci) value of *lcr1*-1 was notably higher than WT, more than 4-fold, indicating a reduced Ci-affinity (Fig. 2A; Supplementary Table S1). Interestingly, the *cah1*-1 mutant showed similar *K*_0.5_ (Ci) values to *lcr1*-1, while the *K*_0.5_ (Ci) value of *lci1*-1 did not significantly differ from WT (Supplementary Fig. S4). Additionally, the *lci1*/*cah1*-1 double mutant exhibited *K*_0.5_ (Ci) values comparable to *cah1*-1 and *lcr1*-1, highlighting a critical role for CAH1 in maintaining Ci affinity under a high HCO_3_^−^/CO_2_ ratio. Unexpectedly, we observed a reduction in HLA3 accumulation in *cah1*-1, *lci1*-1, and *lci1*/*cah1*-1 (Fig. 2B). This reduction in HLA3 accumulation complicates our understanding of the roles of CAH1 and HLA3 in maintaining Ci-affinity. It raises the question of whether the observed decrease in Ci-affinity in *cah1*-1 is solely due to CAH1 loss or if it might also involve a synergistic effect resulting from the simultaneous reduction of both CAH1 and HLA3.

### Acetazolamide's influence on CAH1-mediated Ci-affinity

To further elucidate CAH1's specific contribution to Ci-affinity and separate its effects from those of HLA3, we investigated the response of cells treated with AZA, a CA inhibitor with low membrane permeability, at pH 7.8. Additionally, we generated a *lci1*/*cah1*-1:*CAH1* strain by introducing a gene fragment of *CAH1*, including its putative promoter, 5′-UTR and 3′-UTR, into *lci1*/*cah1*-1 for comparison. In *lci1*/*cah1*-1:*CAH1*, the accumulation levels of CAH1 and HLA3 were increased compared to *lci1*/*cah1*-1 (Fig. 2C). The addition of AZA to WT cells resulted in an increased *K*_0.5_ (Ci) value, aligning with the levels observed in *cah1*-1 and *lci*1/*cah1*-1 mutants (Fig. 2, D and E; Supplementary Table S2). Conversely, when *cah1*-1 and *lci1*/*cah1*-1 mutants were supplemented with bovine CA, their *K*_0.5_ (Ci) value was reduced to WT levels, suggesting that exogenous CA activity can compensate for the loss of CAH1 function by replenishing CO_2_ in the equilibrium. On the other hand, the addition of AZA to *cah1*-1 and *lci1*/*cah1*-1 cells did not cause a further significant increase in *K*_0.5_ (Ci) values. Furthermore, *K*_0.5_ (Ci) values in *lci1*-1/*cah1*:*CAH1* were similar to WT. In combination with previous studies showing a very minor contribution of HLA3 to the maintenance of Ci affinity at pH 7.8 (Yamano et al. 2015), our results demonstrate that the alteration in Ci-affinity observed in *cah1*-1 and *lci1*/*cah1*-1 is primarily attributed to the loss of CAH1 activity. These results affirm the critical function of periplasmic CAH1 in CCM by maintaining high-Ci affinity under CO_2_-limiting conditions.

### Effect of CAH1 mutation on growth rate

**Figure 3.**
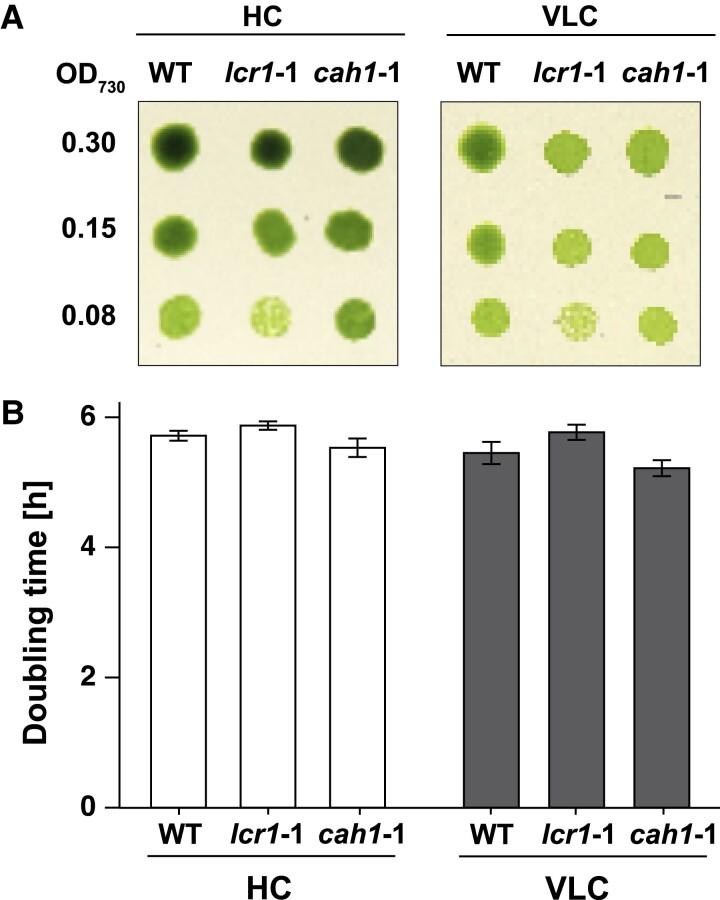
The growth of *lcr1* and *cah1* mutants. **A)** Spot test of WT, *lcr1*-1, and *cah1*-1. Cells were diluted to the indicated optical density (OD_700_ = 0.30, 0.15, or 0.08). Subsequently, 3 *μ*L of the cell suspensions were spotted on agar plates with pH 7.8. The plates were incubated for 4 d under 5% [v/v] CO_2_ (HC) or 0.01% [v/v] CO_2_ (VLC) conditions with continuous light at 120 *μ*mol photons m^−2^ s^−1^. **B)** Doubling time of WT, *lcr1*-1 and *cah1*-1 cells were calculated from three independent experiments. Each cell was cultured in a 5% CO_2_ (HC) or 0.04 CO_2_ (VLC) aeration. Error bars represent standard error (Se).

To further examine the impact of CAH1 deficiency, we evaluated the growth rates of WT, *lcr1*-1, and *cah1*-1 cells. Despite the significant role of CAH1 in maintaining Ci-affinity, no differences in growth rate among these strains were observed under both HC and VLC conditions, as evidenced by spot tests on agar plates at pH 7.8 (Fig. 3A). Additionally, the doubling times for these strains in a liquid medium were comparable (Fig. 3B). These findings suggest that, while CAH1 is crucial for maintaining Ci-affinity, its absence does not impede the overall growth rate under CO_2_-limiting conditions.

## Discussion

In this study, we assessed the Ci-affinity of LCR1-dependent gene mutants created using the CRISPR–Cas9 system across various pH conditions, ranging from acidic to alkaline, to understand their behavior under different environmental scenarios. Notably, at pH 7.8, a condition representative of high HCO_3_^−^/CO_2_ ratios, the *cah1*-1 mutant exhibited a significant decrease in Ci-affinity, highlighting the pivotal role of CAH1 in *Chlamydomonas* cells.

### The function of LCR1 in various environmental stresses

LCR1 is instrumental in the activation of the CCM under CO_2_-limiting conditions, notably regulating the expression of *CAH1* and *LCI1* (Yoshioka et al. 2004). Conversely, under high-light conditions, LCR1 is critical for the expression of *LHCSR3*, essential for photoprotection (Arend et al. 2023). However, our study revealed that LCR1 did not regulate *LHCSR3* expression under CO_2_-limiting conditions (Table 1), demonstrating that the genes controlled by LCR1 vary with environmental context. Our finding that LCR1 does not regulate *LHCSR3* under CO_2_-limiting conditions builds upon our previous work (Yamano et al. 2008), which demonstrated the complex regulation of *LHCSR3* (formerly known as *Li818r-1* and *Li818r-3*). In that study, we showed that *LHCSR3* is induced by high-light in a CCM1-independent manner, while under CO_2_-limiting conditions, its expression is CCM1-dependent. The current results, showing that LCR1 (a downstream factor of CCM1) does not regulate *LHCSR3* under CO_2_-limiting conditions, suggest a more intricate regulatory network. This implies that while CCM1 is involved in *LHCSR3* regulation under CO_2_-limiting conditions, it likely acts through factors other than LCR1.

To further elucidate the regulatory mechanism of *LHCSR3* expression and the roles of CCM1 and LCR1 in this process, future studies should investigate the expression patterns of *LHCSR3* under various combinations of light intensity and CO_2_ availability. Additionally, identifying transcription factors that mediate CCM1-dependent *LHCSR3* expression under CO_2_-limiting conditions would be crucial. Exploring potential interactions between LCR1 and other transcription factors involved in *LHCSR3* regulation could also provide valuable insights. These investigations could reveal how *Chlamydomonas* fine-tunes its gene expression in response to complex environmental changes, particularly in the context of carbon concentration mechanisms and photoprotection. Such findings underscore the importance of phenotypic analysis under various environmental conditions. Further insights into the diverse functions of transcription factors are expected from the recent large-scale systematic analysis (Fauser et al. 2022), which examines mutant phenotypes under various environmental growth conditions and chemical treatments.

### CAH1 facilitates indirect HCO_3_^−^ utilization under alkaline conditions

**Figure 4.**
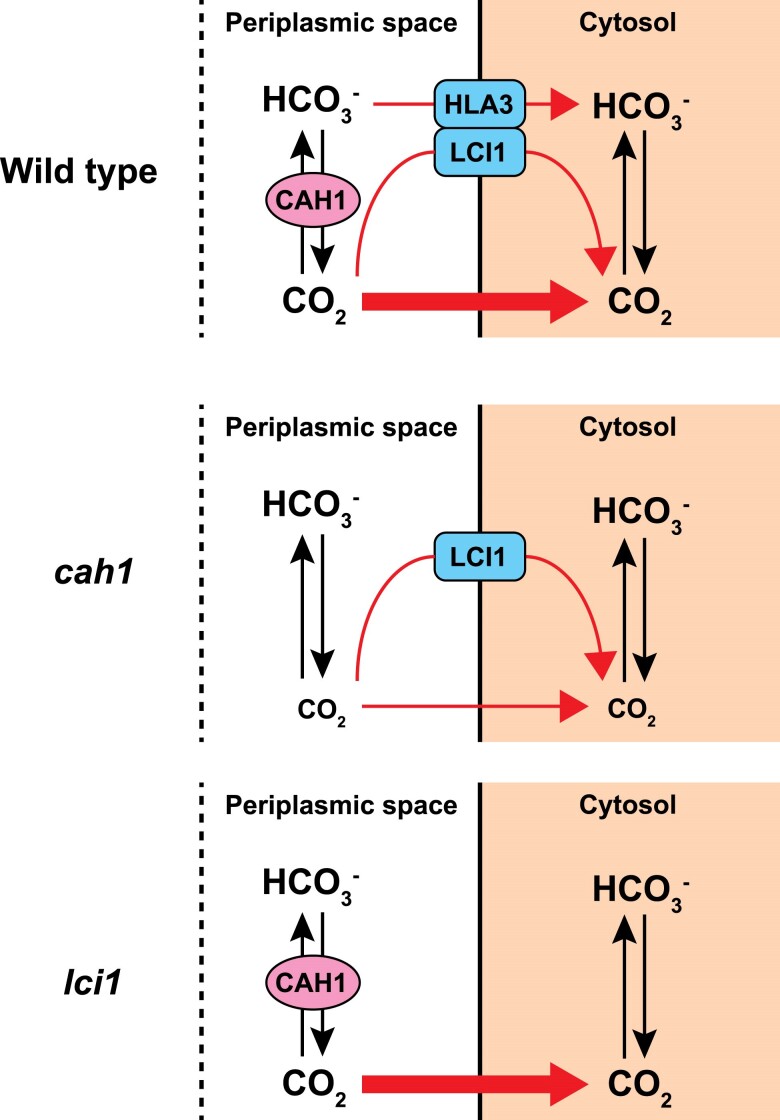
Models for Ci uptake pathway in WT, *cah1* and *lci1* mutants. Tentative models show how WT, *cah1*, and *lci1* mutants uptake Ci across the plasma membrane under CO_2_-limiting conditions and at pH 7.8. Black arrows indicate the interconversion between CO_2_ and HCO_3_^−^. Red arrows show the Ci uptake pathway from the periplasmic space into the cytosol.

We demonstrated that CAH1 is crucial for maintaining high Ci-affinity in *Chlamydomonas* WT cells under alkaline conditions (pH 7.8), supporting the contribution of CAH1 to indirect utilization of abundant HCO_3_^−^ (Fig. 4). This aligns with previous reports indicating enhanced transcription, protein accumulation, and CA activity of CAH1 at higher pH levels (Fett and Coleman 1994). An earlier study did not reveal significant differences in Ci-affinity between WT and *cah1* mutants (Van and Spalding 1999), possibly due to the measurements performed at neutral pH. In addition, our research utilized a consistent parental strain for *cah1* mutants, ensuring a more accurate evaluation of CAH1's impact.

Whole genome sequencing of various *Chlamydomonas* laboratory strains has revealed genetic diversity among these strains (Gallaher et al. 2015). Furthermore, we have previously reported results suggesting that WT strains can acquire characteristics during long-term subculturing (Tsuji et al. 2023). These findings underscore the potential for genetic drift and the accumulation of spontaneous mutations in laboratory strains over time. As demonstrated in our previous studies (Toyokawa et al. 2020; Tsuji et al. 2023), this study reaffirms the importance of using mutants generated from the same parental strain for accurate phenotypic analysis in *Chlamydomonas* reverse genetics. By using the C9 strain as the common background for all our mutants, we minimize the confounding effects of strain-specific genetic variations, ensuring more reliable and reproducible results.

In *Chlamydomonas*, periplasmic CA was identified about four decades ago (Kimpel et al. 1983), and physiological experiments using weakly permeable sulfonamide inhibitors established the well-known model that periplasmic CA facilitates the indirect utilization of bulk HCO_3_^−^ (Moroney et al. 1985, Aizawa and Miyachi 1986). Although the contradictory result in the previous analysis of *cah1* mutant (Van and Spalding 1999) had raised controversy about the function of periplasmic CA, we demonstrated the importance of CAH1 at alkaline conditions, strengthening the original hypothesis that periplasmic CA supplies CO_2_ from HCO_3_^−^. Regarding catalytic direction (hydration or dehydration), there is an opposing hypothesis based on a mathematical modeling, in which periplasmic CA recaptures CO_2_ leaked from the cell through the hydration (Fridlyand 1997). However, this hypothesis is unlikely in *Chlamydomonas* because (i) analysis using membrane inlet mass spectrometry (MIMS) detected net CO_2_ uptake, but not CO_2_ efflux, by the cell when external CA was inhibited or removed (Shiraiwa et al. 1993; Sültemeyer et al. 1989), and (ii) light-dependent alkalization of medium was observed (Shiraiwa et al. 1993). These physiological measurements were performed at pH 8.0, which is similar to the conditions (pH 7.8) where our *cah1* mutant displayed lower Ci-affinity than the parental strain (Fig. 2A). Importance of periplasmic CA was also suggested by the pronounced inhibitory effect of weakly permeable sulfonamide inhibitor at alkaline pH (pH 8.0) (Moroney et al. 1985). Thus, the long-standing discrepancy between physiological and genetic evidence has been solved, and both approaches provide consistent support for the original model that periplasmic CA enhances indirect HCO_3_^−^ utilization by accelerating dehydration. Besides *Chlamydomonas*, the enhanced CO_2_ uptake by periplasmic CA-mediated dehydration is supported in various algal species. In the relatively distant green alga *Chlorella*, physiological studies have provided evidence for the role of periplasmic CA (Matsuda et al. 1999). Furthermore, the enhanced CO_2_ uptake by periplasmic CA-mediated dehydration is also supported in some marine diatoms such as *T. pseudonana* and *O. sinensis* by kinetic analysis of CO_2_ uptake using MIMS and direct measurement of cell surface pH changes, respectively (Hopkinson et al. 2013; Chrachri et al. 2018), suggesting the generality of the classical model in diverse algal groups. Notably, CAH1 in *Chlamydomonas* is α-type while diatoms have δ- and ζ-type in the periplasmic space (Samukawa et al. 2014), suggesting the convergent evolution of the CCM in different lineages as previously discussed (Matsuda et al. 2017).

### Multiple strategies of Ci-uptake in *Chlamydomonas*

In *Chlamydomonas*, Ci uptake across the plasma membrane involves multiple transport strategies. These include direct pathways of CO_2_ through LCI1 and HCO_3_^−^ through HLA3, respectively, along with indirect pathways involving CAH1 (Fig. 4). Despite no significant reduction in Ci affinity in *lci1*-1 mutants (Fig. 2A;Supplementary Table S1), LCI1's cooperative role with other channels cannot be ruled out. This unexpected result suggests a complex CO_2_ uptake mechanism in *Chlamydomonas*. It is possible that unidentified CO_2_ channels or transporters may compensate for the loss of LCI1. Additionally, functional redundancy in the Ci uptake system might allow other pathways to compensate for the deficiency of a single gene. Further analysis, such as creating multiple gene knockout mutants, could help elucidate the intricate nature of this Ci uptake system and the specific role of LCI1 within it. Additionally, post-translational modifications of LCI1 could play a crucial role in its function or regulation. Future studies investigating these aspects, including the identification of potential LCI1 interacting partners and analysis of its post-translational modifications, will be essential to fully understand the role of LCI1 in the CO_2_ uptake mechanism of *Chlamydomonas*.

Interestingly, the accumulation level of HLA3 was reduced in *cah1*-1 and *lci1*-1 (Fig. 4). The complete loss of CAH1 and LCI1 may have caused this phenotype, as HLA3 accumulation was not altered in *lcr1*-1, which still expresses low levels of *CAH1* and *LCI1* (Figs 1 and 2B; Supplementary Data Set 1). Since HLA3 and LCI1 interact and form a complex on the plasma membrane (Mackinder et al. 2017), it is possible that the formation of the HLA3–LCI1 complex was inhibited in *lci1*-1. Furthermore, the expression of *HLA3* is closely tied to that of LCIA, a HCO_3_^−^ transporter located on the chloroplast envelope (Yamano et al. 2015). This multitiered control of HLA3 expression by various factors, including CAH1, LCI1, and LCIA, suggests a complex regulatory network governing Ci uptake. To further unravel the complexities of this regulatory network, future studies should investigate the physical interaction between CAH1 and HLA3, as well as the impact of LCIA, LCI1, and CAH1 deficiency on *HLA3* expression.

Notably, from our findings that *cah1*-1 mutants displayed a substantially higher *K*_0.5_ (Ci) value compared to WT under pH 7.8 conditions (Fig. 2A;Supplementary Table S1), emphasizing CAH1's primary role in Ci uptake into chloroplasts at this pH conditions (Fig. 4). Despite the reduced Ci-affinity, the absence of growth rate differences among *lcr1*-1, *cah1*-1, and WT under CO_2_-limiting conditions (Fig. 3, A and B) suggests that the CO_2_ concentrations used in our growth experiments were still sufficient to support normal growth in the mutants. This observation raises the possibility that the growth conditions used in this study may not have been optimal for detecting the effects of CAH1 loss on growth rate. Future studies exploring a wider range of CO_2_ concentrations and pH conditions are needed to fully elucidate the impact of CAH1 deficiency on growth under varying environmental conditions.

### Diversity of periplasmic CA functions in *Chlamydomonas*

Our findings reveal that AZA significantly reduced Ci-affinity in cells, aligning with previous research (Moroney et al. 1985). Besides CAH1, CAH2 and CAH8 are also located in the periplasmic space (Moroney et al. 2011). Although CAH2 shares a similar amino acid sequence with CAH1, its expression is induced under high CO_2_ conditions, differing from CAH1 (Fujiwara et al. 1990). CAH8, a β-type CA with a transmembrane domain, is positioned closer to the plasma membrane than CAH1 under varying CO_2_ conditions (Ynalvez et al. 2008). The comparable Ci-affinity in AZA-treated WT cells and *cah1* mutant underscores CAH1's greater role in Ci uptake under CO_2_-limiting conditions compared to CAH2 and CAH8. Future studies focusing on the regulation of these periplasmic CAs and their compensatory interactions are essential, potentially informing bioengineering approaches to enhance microalgae photosynthesis.

## Materials and methods

### Chlamydomonas (*C. reinhardtii*) strains and cultural conditions

The WT strain C9, obtained from the IAM Culture Collection at the University of Tokyo, was utilized for physiological and biochemical experiments. Strain C9 is now available from the Microbial Culture Collection at the National Institute for Environmental Studies, Japan, as strain NIES-2235 (alternatively named CC-5098 in the *Chlamydomonas* Resource Center). The cells were precultured in a TAP medium and subsequently resuspended in 50 mL of MOPS-*P* medium. They were grown under a 5% (v/v) CO_2_ atmosphere with a light intensity set at 120 *μ*mol photons m^−2^ s^−1^, following the method described by Toyokawa et al. (2020), until they reached the mid-logarithmic phase of growth. For the induction of VLC conditions, cells acclimated to high-CO_2_ conditions were centrifuged, resuspended in fresh MOPS-P medium, and then cultured with air bubbling containing 0.04% (v/v) CO_2_ at the same light intensity.

### Measurement of photosynthetic O_2_-evolving activity

Cells were harvested and resuspended in Ci-depleted 20 mm MES–NaOH (pH 6.2), MOPS–NaOH (pH 7.0), or HEPES–NaOH (pH 7.8) buffers, adjusting the density to 10 to 20 *μ*g chlorophyll per milliliter. The photosynthetic oxygen evolution rate was then measured using a Clark-type oxygen electrode (Hansatech Instruments) as described previously (Yamano et al. 2008). AZA adjusted to a concentration of 5 mm and dissolved in DMSO, was added to the measuring buffer at a 1% [v/v]. Bovine CA was added into the buffer, achieving a concentration of 2.0 *μ*g mL^–1^. For comparison, 1% DMSO was introduced to samples without AZA.

### Generation of mutants by the CRISPR–Cas9 system

For CRISPR–Cas9-mediated genome editing, guide RNAs were designed using the CRISPOR tool (Concordet and Haeussler 2018), as detailed in Supplementary Figs. S1 to S3. The introduction of the ribonucleoprotein complex and the *AphVII* or *AphVIII* cassette into cells followed the method of Tsuji et al. (2023). Primer sets used for screening are shown in Supplementary Figs. S1 to S3, and their sequences are listed in Supplementary Table S3.

### Immunoblotting analysis

Total protein extraction, SDS–polyacrylamide gel electrophoresis (SDS/PAGE), and immunoblotting analyses were carried out as previously described (Wang et al. 2016). Primary antibodies were utilized at the following indicated dilutions: anti-HLA3 at 1:1,250, anti-LCIA at 1:5,000, anti-LCI1 at 1:5,000, anti-LCIB at 1:5,000, anti-CAH1 at 1:2,500, anti-CAH3 at 1:2,000, anti-CCM1 at 1:2,500, and anti-Histone H3 at 1:10,000. A horseradish peroxidase-conjugated goat anti-rabbit IgG antibody from Life Technologies was employed as the secondary antibody at a dilution of 1:10,000 to detect the primary antibodies.

### RNA-seq analysis

Total RNA was extracted from cells using the RNeasy Plant Mini Kit (QIAGEN), following the manufacturer's instructions. After RNA purification, the total RNA was analyzed using the Illumina Novaseq 6000 system. In each condition, sequencing data were obtained from two biological replicates. The resulting reads were aligned with version 5.6 of the *C. reinhardtii* genome annotation, which was downloaded from https://phytozome-next.jgi.doe.gov/. The alignment, counting of reads, and normalization of read counts were performed according to the methods previously described in Shimamura et al. (2023).

### Accession numbers

The accession numbers of the Phytozome database for *Chlamydomonas* genes *LCR1*, *CAH1*, *LCI1*, and *LCI6* are *Cre09.g399552*, *Cre04.g223100*, *Cre03.g162800*, and *Cre12.g553350*, respectively.

## Supplementary Material

kiae463_Supplementary_Data

## Data Availability

Data deposition: The RNA-seq raw data in this paper have been deposited in the DNA Data Bank of Japan (DDBJ) Sequence Read Archive (DRA) (accession no. DRA017670).

## Author contribitions

T.Y. and H.F. conceived and designed the study; D.S. performed most of the experiments; T.I. and A.M. contributed to mutant isolation; K.M. provided additional supervision and resources; D.S., Y.T., and T.Y. wrote the article, and all authors approved it. T.Y. agreed to serve as the author responsible for contact and ensure communication.

## Supplementary data

The following materials are available in the online version of this article.

**Supplementary Figure S1.** The *lcr1* mutant generated by the CRISPR–Cas9 system.

**Supplementary Figure S2.** The mutants of *lci6* and *Cre10.g426800* generated by the CRISPR–Cas9 system.

**Supplementary Figure S3.** The mutants of *cah1* and *lci1* generated by the CRISPR–Cas9 system.

**Supplementary Figure S4.** Oxygen-evolving activity of WT and transformant cells in response to external dissolved Ci concentrations.

**Supplementary Table S1.** Photosynthetic parameters of WT and transformant cells.

**Supplementary Table S2.** Effect of AZA and bovine CA on photosynthetic parameters of WT and *cah1*-1 cells.

**Supplementary Table S3.** Sequences of primers used in this study.

## Dive Curated Terms

The following phenotypic, genotypic, and functional terms are of significance to the work described in this paper:

LCR1 Gramene: AT5G48543
LCR1 Araport: AT5G48543
