# Supplementary material for: Periplasmic carbonic anhydrase CAH1 contributes to high inorganic carbon affinity in Chlamydomonas reinhardtii
Source: Plant Physiol. 2024 Aug 30;196(4):2395–404. doi: 10.1093/plphys/kiae463 (PMC11637766; doi:10.1093/plphys/kiae463)
Supplement: kiae463_Supplementary_Data [file kiae463_supplementary_data.zip › Supplementary figures.pdf]

Supplementary Data

Periplasmic carbonic anhydrase CAH1 contributes to high inorganic carbon affinity in *Chlamydomonas reinhardtii*

Daisuke Shimamura[1,3], Tomoaki Ikeuchi[1], Ami Matsuda[1], Yoshinori Tsuji[1], Hideya Fukuzawa[1, 2], Keiichi Mochida[3], Takashi Yamano[1, *]
[1]Graduate School of Biostudies, Kyoto University, Kyoto, Japan
[2]Senior author
[3]RIKEN Center for Sustainable Resource Science, Yokohama, Japan
[4]Center for Living Systems Information Science (CeLiSIS), Kyoto University, Kyoto, Japan
*Author for communications: tyamano@lif.kyoto-u.ac.jp (T. Y.)

Supplemental Figure S1 The *lcr1* mutant generated by the CRISPR-Cas9 system
Supplemental Figure S2 The mutants of *lci6* and *Cre10.g426800* generated by the CRISPR-Cas9 system
Supplemental Figure S3 The mutants of *cah1* and *lci1* generated by the CRISPR-Cas9 system
Supplemental Figure S4 Oxygen-evolving activity of wild type (WT) and transformant cells in response to external dissolved Ci concentrations
Supplemental Table S1 Photosynthetic parameters of wild type (WT) and transformant cells
Supplemental Table S2 Effect of AZA and CA on photosynthetic parameters of wild type (WT) and transformant cells
Supplemental Table S3 Sequences of primers used in this study

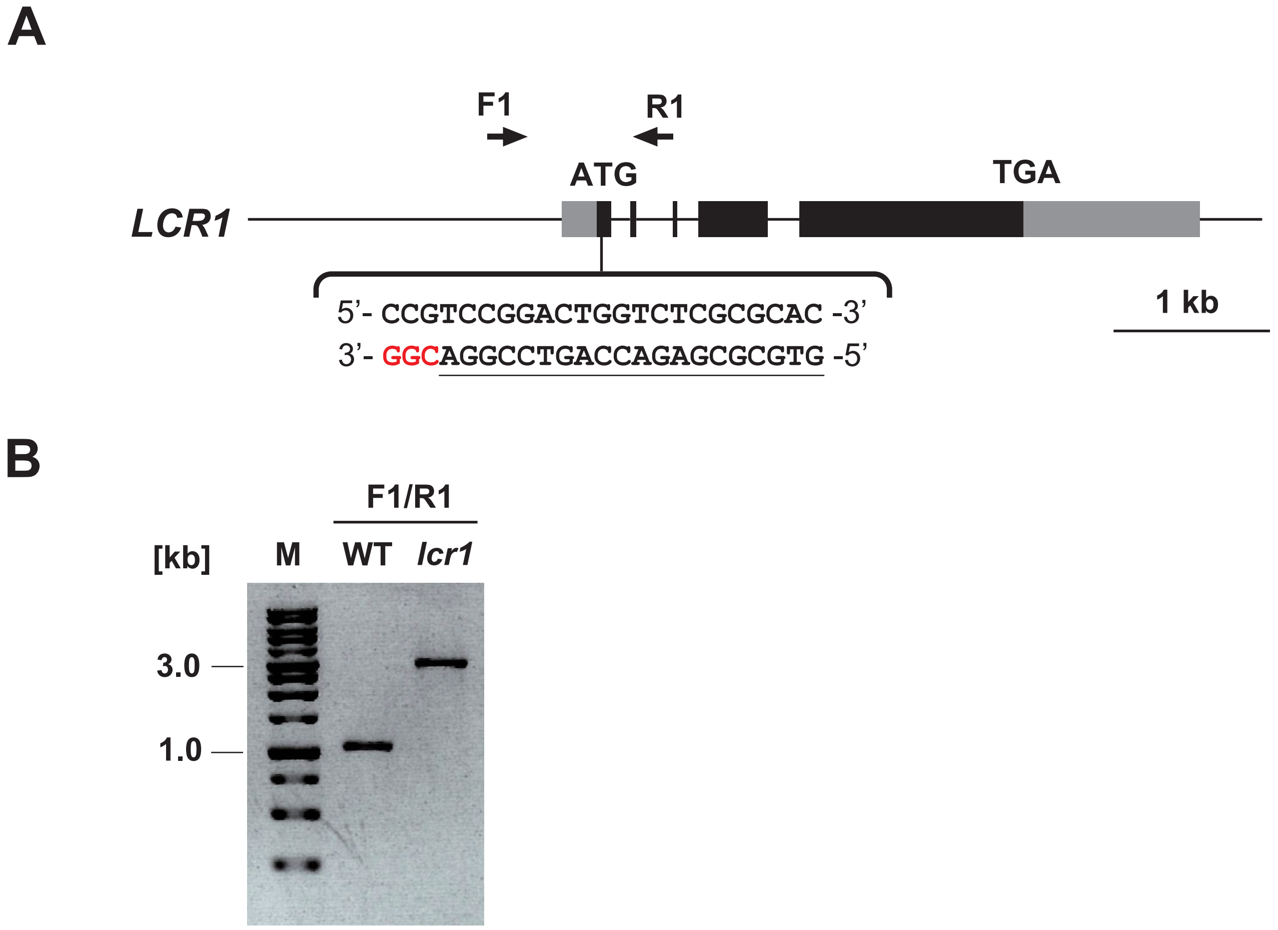

Supplementary Figure S1. The *lcr1* mutant generated by the CRISPR-Cas9 system

A, The position of guide RNA (gRNA) and protospacer adjacent motif (PAM) sequence in the first exon of *LCR1* gene. gRNA sequences are underlined and PAM sequences are in red letters. The solid rectangles indicate the exons of *LCR1* gene. Arrows indicate primers utilized for PCR screening. B, Genomic PCR to confirm insertion of the *AphVII* cassette in LCR1 gene. Wild type is abbreviated as WT. M represents the DNA marker lane.

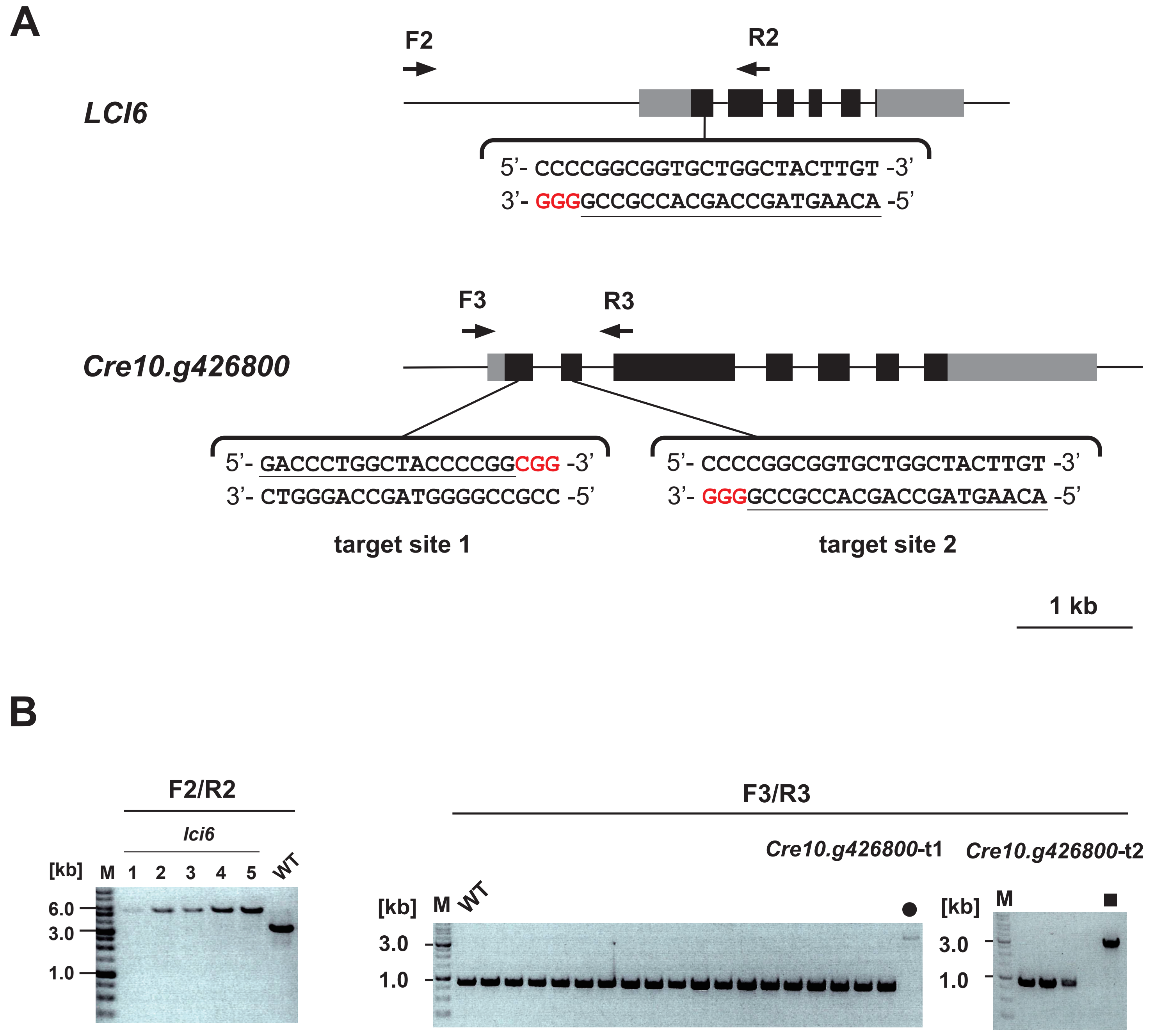

Supplementary Figure S2. The mutants of *lci6* and *Cre10.g426800* generated by the CRISPR-Cas9 system

A, The positions of guide RNA (gRNA) and protospacer adjacent motif (PAM) sequence in *LCI6* and *Cre10.g426800* gene. gRNA sequences are underlined and PAM sequences are in red letters. The solid rectangles indicate the exons of *LCR1* gene. Arrows indicate primers utilized for PCR screening. B, Genomic PCR to confirm insertion of the *AphVII* cassette in *LCI6* and *Cre10.g426800* gene. The circle and square symbols indicate insertional mutations at target site 1 and target site 2 in *Cre10.g426800* gene, respectively. Wild type is abbreviated as WT. M represents the DNA marker lane.

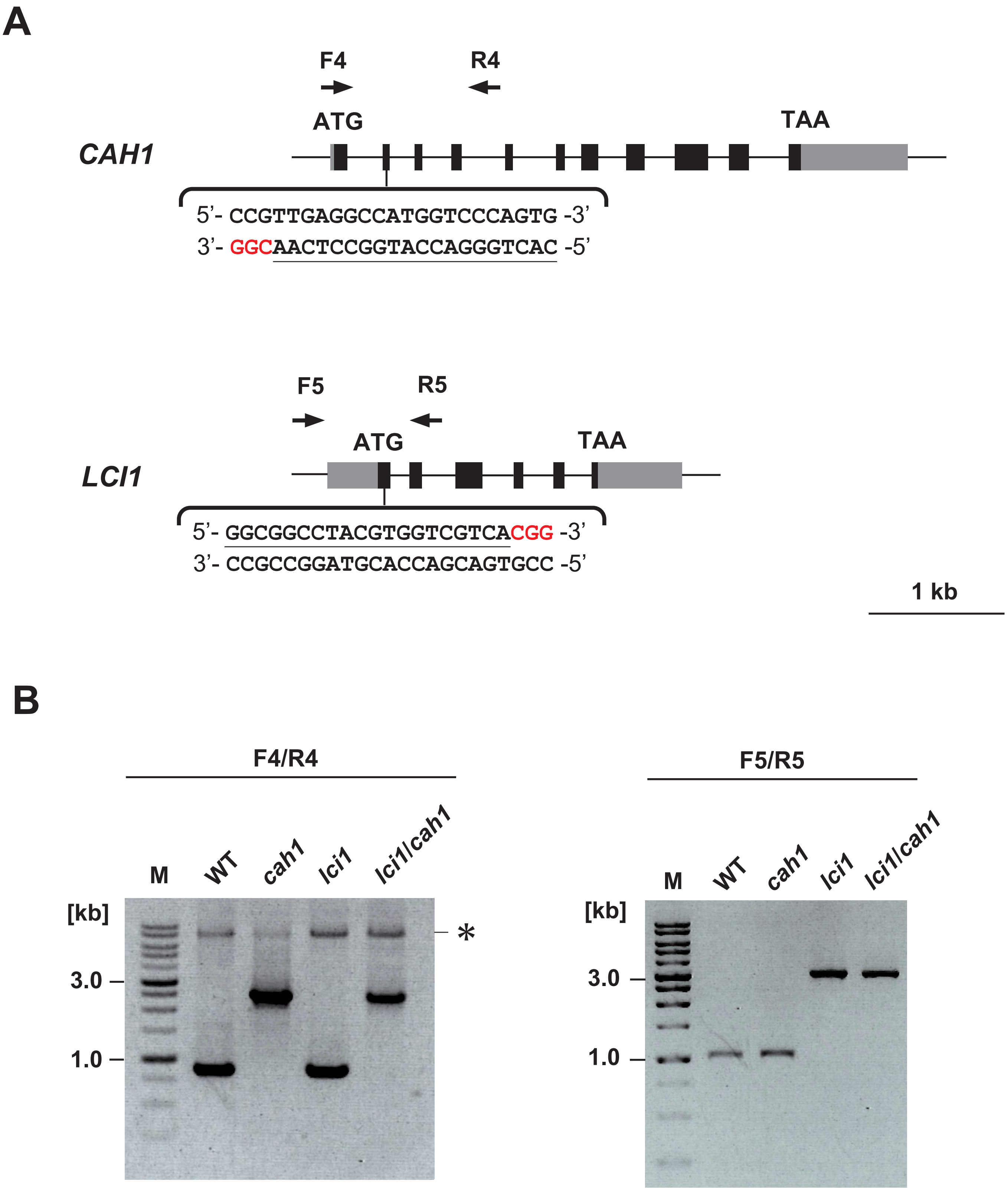

Supplementary Figure S3 The mutants of *cah1* and *lci1* generated by the CRISPR-Cas9 system
A, The positions of guide RNA (gRNA) and protospacer adjacent motif (PAM) sequence in *CAH1* and *LCI1* gene. gRNA sequences are underlined and PAM sequences are in red letters. The solid rectangles indicate the exons of *CAH1* and *LCI1* gene. Arrows indicate primers utilized for PCR screening. B, Genomic PCR to confirm insertion of the *AphVIII* and *AphVII* cassette in *CAH1* and *LCI1* gene. An asterisk indicates a non-specific band. Wild type is abbreviated as WT. M represents the DNA marker lane.

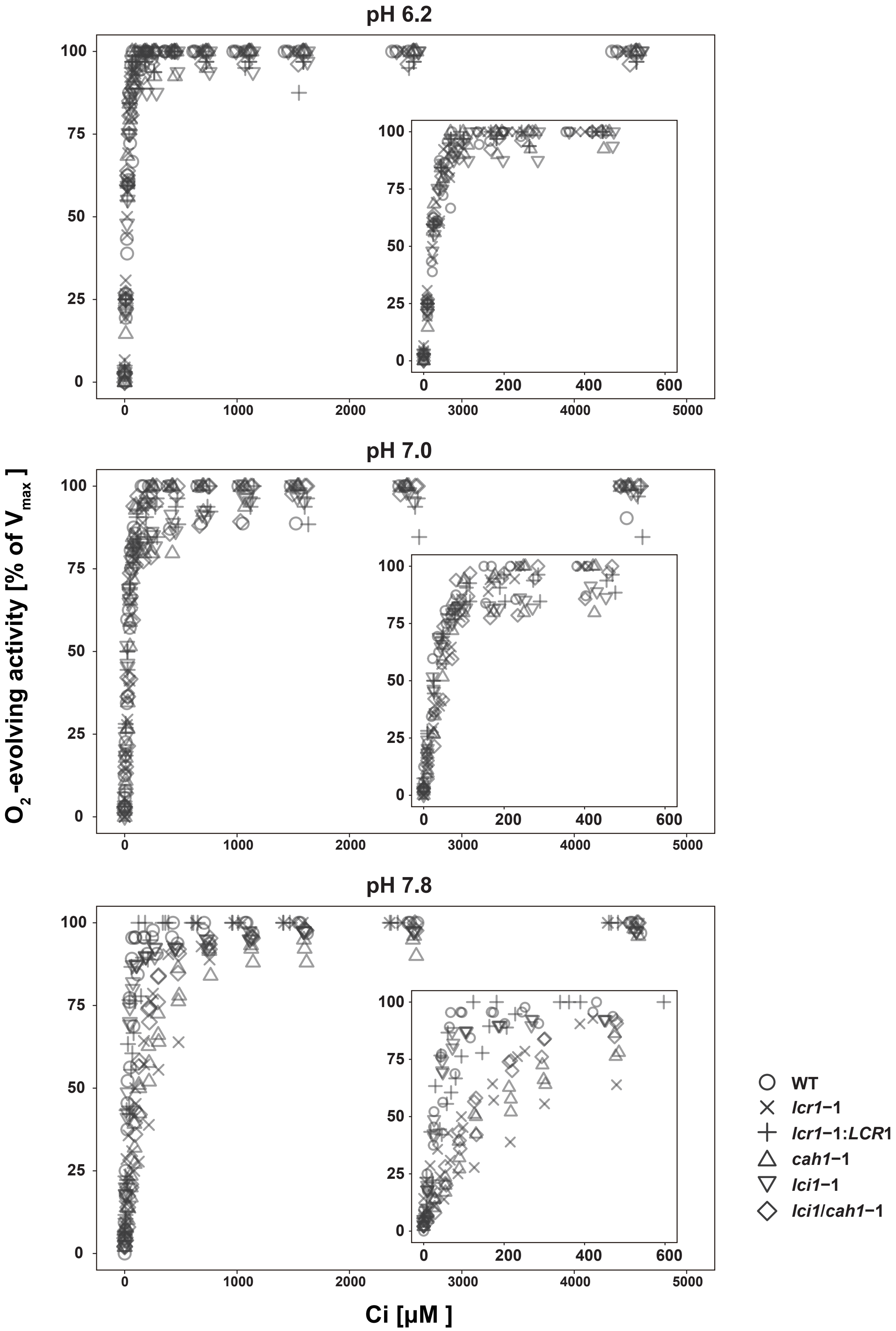

Supplementary Figure S4 Oxygen-evolving activity of wild type (WT) and transformant cells in response to external dissolved Ci concentrations

Responses of net O_2-evolving activities of WT (open circle), *lcr1*-1 (cross), *lcr1*-1:*LCR1* (plus), *cah1*-1 (open triangle), *lci1*-1 (open inverted triangle) and *lci1*/*cah1*-1(open diamond) against calculated CO_2 concentrations at pH 6.2, 7.0 and 7.8. Before measurements, cells were grown in the liquid culture aerated with 0.04% CO_2 for 12 h at pH 7.0. Insets show plots at external dissolved Ci concentrations below 600 µM.
