# Supplementary material for: Periplasmic carbonic anhydrase CAH1 contributes to high inorganic carbon affinity in Chlamydomonas reinhardtii
Source: Plant Physiol. 2024 Aug 30;196(4):2395–404. doi: 10.1093/plphys/kiae463 (PMC11637766; doi:10.1093/plphys/kiae463)
Supplement: kiae463_Supplementary_Data [file kiae463_supplementary_data.zip › Supplementary tables.pdf]

Supplementary Data

Periplasmic carbonic anhydrase CAH1 contributes to high inorganic carbon affinity in *Chlamydomonas reinhardtii*

Daisuke Shimamura[1,3], Tomoaki Ikeuchi[1], Ami Matsuda[1], Yoshinori Tsuji[1], Hideya Fukuzawa[1, 2], Keiichi Mochida[3], Takashi Yamano[1, *]
[1]Graduate School of Biostudies, Kyoto University, Kyoto, Japan
[2]Senior author
[3]RIKEN Center for Sustainable Resource Science, Yokohama, Japan
[4]Center for Living Systems Information Science (CeLiSIS), Kyoto University, Kyoto, Japan
*Author for communications: tyamano@lif.kyoto-u.ac.jp (T. Y.)

Supplemental Figure S1 The *lcr1* mutant generated by the CRISPR-Cas9 system
Supplemental Figure S2 The mutants of *lci6* and *Cre10.g426800* generated by the CRISPR-Cas9 system
Supplemental Figure S3 The mutants of *cah1* and *lci1* generated by the CRISPR-Cas9 system
Supplemental Figure S4 Oxygen-evolving activity of wild type (WT) and transformant cells in response to external dissolved Ci concentrations
Supplemental Table S1 Photosynthetic parameters of wild type (WT) and transformant cells
Supplemental Table S2 Effect of AZA and CA on photosynthetic parameters of wild type (WT) and transformant cells
Supplemental Table S3 Sequences of primers used in this study

Supplemental Table S1. Photosynthetic parameters of wild type (WT) and transformant cells

pH	Strain name	V_{max} of O_2 -evolving activity [μmol O_2 mgChl^{-1} h^{-1}]	$K_{0.5}$ (Ci) [μM]
6.2	WT	218 ± 25	25 ± 3
	lcr1 -1	178 ± 15	25 ± 4
	lcr1-1:LCR1	180 ± 7	21 ± 2
	cah1 -1	228 ± 10	24 ± 3
	lci1 -1	230 ± 11	25 ± 1
	lci1 /*cah1* -1	263 ± 8	22 ± 1
7.0	WT	215 ± 18	30 ± 5
	lcr1 -1	191 ± 20	48 ± 4
	lcr1-1:LCR1	182 ± 10	29 ± 2
	cah1 -1	272 ± 3	43 ± 3
	lci1 -1	297 ± 6	29 ± 2
	lci1 /*cah1* -1	231 ± 23	43 ± 8
7.8	WT	205 ± 10	32 ± 4
	lcr1 -1	239 ± 18	168 ± 52
	lcr1-1:LCR1	251 ± 33	42 ± 10
	cah1 -1	171 ± 9	155 ± 12
	lci1 -1	179 ± 2	31 ± 1
	lci1 /*cah1* -1	170 ± 3	134 ± 8
	lci6 -1	300 ± 118	34 ± 4
	lci6 -2	241 ± 35	33 ± 5
	lci6-3	272 ± 60	27 ± 3
	Cre10.g426800 -1	210 ± 25	21 ± 2
	Cre10.g426800 -2	246 ± 86	22 ± 7

Cells grown in 5% CO_2 were shifted to 0.04% CO_2 for 24 h at pH 7.0. The data are shown ± standard error (SE), which was obtained from three biological replicates. V_{max}, maximum O_2-evolving activity; $K_{0.5}$ (Ci), Ci concentration required for half of V_{max}.

Supplemental Table S2. Effect of acetazolamide (AZA) and CA on photosynthetic parameters of wild type (WT) and transformant cells

pH	Strain name and measurement condition	V_{max} of O_2 -evolving activity [µmol O_2 mgChl^{-1} h^{-1}]	$K_{0.5}$ (Ci) [µM]
7.8	WT Mock	176 ± 14	51 ± 4
	WT + 50 µM AZA	193 ± 9	178 ± 22
	cah1-1 Mock	215 ± 5	126 ± 9
	cah1-1 + 50 µM AZA	199 ± 2	164 ± 37
	cah1-1 + 2.0 µg/mL CA	195 ± 9	46 ± 6
	lci1/*cah1*-1 Mock	194 ± 6	137 ± 2
	lci1/*cah1*-1 + 50 µM AZA	199 ± 2	129 ± 1
	lci1/*cah1*-1 + 2.0 µg/mL CA	195 ± 14	47 ± 4
	lci1/*cah1*-1:*CAH1* Mock	179 ± 7	48 ± 2

Cells grown in 5% CO_2 were shifted to 0.04% CO_2 for 24 h at pH 7.0. The data are shown ± standard error (SE), which was obtained from three biological replicates. V_{max}, maximum O_2-evolving activity; $K_{0.5}$ (Ci), Ci concentration required for half of V_{max}. In experiment with AZA or bovine CA, 1% (v/v) DMSO was used as a mock.

Supplemental Table S3. Sequences of primers used in this study

Primer name	Sequence (5' to 3' direction)
F1	ATACACCAAAATCTATTCGCCTTGAAAC
R1	CCCACTGACACACTGCTACTGC
F2	GAAGGAGAGTGGCGTCAAGTACAAG
R2	CAATGATCAGTGTTCTTGGCAATTC
F3	CCTGCTAAATTTCCTATCACAAGCTAAC
R3	TCGTGTGTGTGTGTATATAAAGCTTGGA
F4	GGCTAACCGACTCGAACAATGAACCAAGAACAAGC
R4	CACGTGGCGCGACACGCTGCCTCCC
F5	GTCAGCTTGTAGCACTACGCTAGTTGTC
R5	ATACTAGCTCACCGTTGATGTTGTCCAT
