# Supplementary material for: Periplasmic carbonic anhydrase CAH1 contributes to high inorganic carbon affinity in Chlamydomonas reinhardtii
Source: Plant Physiol. 2024 Aug 30;196(4):2395–404. doi: 10.1093/plphys/kiae463 (PMC11637766; doi:10.1093/plphys/kiae463)
Supplement: kiae463_Supplementary_Data [file pp.00341.2024-s07.pdf › pp.00341.2024-s07.pdf]

Plant Physiology®

Periplasmic carbonic anhydrase CAH1 contributes to high inorganic carbon affinity in Chlamydomonas reinhardtii

Daisuke Shimamura, Tomoaki Ikeuchi, Ami Matsuda, Yoshinori Tsuji, Hideya Fukuzawa, Keiichi Mochida, and Takashi Yamano

Review Timeline:

Submission Date:	04-Mar-2024
Editorial Decision:	02-Apr-2024
Revision Received:	19-Jul-2024
Accepted:	30-Jul-2024

02-Apr-2024

Dr. Takashi Yamano
Kyoto Daigaku
Kyoto 606-8502
Japan

RE: Periplasmic carbonic anhydrase CAH1 contributes to high inorganic carbon affinity in Chlamydomonas reinhardtii

Dear Prof. Yamano:

We have received reviews of your manuscript, which you may view at the end of this letter. On the basis of the advice received and my own evaluation, I believe that, pending revision, this article may ultimately be acceptable for publication in Plant Physiology.

Both reviewers felt that the work was solid and the data clearly presented. However there was some concern over novelty, particularly from reviewer 2. Following consultation, both reviewers felt that the two experiments suggested by reviewer 1 could help to address these concerns. Please could you consider these and respond to the comments raised by both reviewers.

When you are ready to submit your revision, click the below link and find the red arrow under your Author Tasks heading for this revision submission.

If you want to visit this version of the manuscript, you may click the link below and then click on the folder that reads "Post Decision Manuscripts".

Link Not Available

When uploading the revised version of this article, please be sure to include as complete an answer as possible to the referees' criticisms during the first round of reviews in the "response to reviewers" section.

Plant Physiology considers a revision for up to 60 days after a manuscript has been provisionally accepted; limited extension of this timeframe is at the discretion of the Monitoring Editor. A reminder notice will be sent to you 2 weeks before the deadline, at which time you may choose to contact the editorial office to request an extension; if so, please provide a justification for your request. After the expiration of this deadline, the article would need to be submitted as a resubmission and would be handled as such.

Thank you very much for giving us an opportunity to review this work. I look forward to receiving the next version.

Sincerely,

Editor, Plant Physiology

Want to add this revision deadline to your calendar? Click below!

-- Reviewer comments:
Reviewer #1 (Comments for the Author):

Shimamura et al. use a CRISPR-Cas9 approach to address a long debated question in the Chlamydomonas CCM literature; the potential role of the periplasmic carbonic anhydrase, CAH1. By utilising the same C9 background strain for all experiments, the authors allow direct comparison between the strains, which has been a limitation in previous studies. By generating lcr1, lci1, cah1 and lci1/cah1 CRISPR lines, the authors convincingly show that CAH1, under the control of LCR1, is the major downstream factor for Ci affinity under limiting CO_2 conditions and elevated pH that is controlled by LCR1.

Although the study is mostly robust throughout, two additional experiments would address the only major concern and a more minor concern I have with the manuscript.

1) As the authors state, the cah1-1 mutant displays reduced Ci affinity that can be rescued by the addition of extracellular carbonic anhydrase. However, the lack of HLA3 in the cah1-1 mutant does not allow the authors to completely decouple the action of HLA3 and CAH1 at membrane, despite the CA inhibition experiments they present. Complementation of the cah1-1 (and ideally the lci1/cah1-1) mutant and analysis of HLA3 presence and Ci affinity (and optionally growth phenotype) would allow a more convincing conclusion of this body of work.

2) Secondly, much of the previous debate around CAH1's role has to do with conclusions from the use of CA inhibitors (acetazolamide). In this study, the authors leverage the same approach to conclude that CAH1 inhibition in the periplasm has the same effect as CAH1 knockout in the cah1-1 line. In these experiments, the same acetazolamide treatment should be applied to the cah1-1 line (and optionally the cah1-1 supplemented with bovine CA experiment) to show that acetazolamide treatment does not further reduced Ci, which would be indicative of membrane permeability and the intracellular inhibition proposed by Williams and Turpin (1987).

Several other small additions are requested, but do not require additional experimentation:
- The complete RNA-seq data (including downregulated genes) for the lcr1 and C-LCR1 lines should be included in Supplemental Dataset 1 to allow the reader to derive the values in Table 1.
- Details of how the complementation of lcr1 was completed are missing. The promoter, UTRs and terminators used in this complementation are important to the complementation.
- Details of the growth conditions (e.g. pH) for experiments are missing for the following experiments:
o RNA-seq - which pH, which media?
o Fig. 1 - which pH?
o Fig. 2b - which pH?
- The oxygen evolution curves used to derive values in Figs. 2a, 2d and Supplemental Tables S1 and S2 should be provided in the supplementary data.
- Additions to the methods are required in several places, as outlined below:
o Photosynthetic O2-evolving activity methods should at least cite a previous publication in which the same approach was used. There is an error in the title as well.
o The CRISPR reference Tsuji et al., 2022 doesn't exist - this should be 2023.
o Immunoblotting should cite the original reference (Wang et al., 2016, PNAS) which is cited in Shimamura et al., (2023).
o For the RNA-seq experiments, were replicates used?
o Although the CA inhibitor details are in the figure legend, it would be useful to have the details of these experiments in the methods section.
- More discission as to why there is no phenotype are required. This is an important discrepancy between the Ci affinity and expected phenotype.

Minor line-by-line comments listed below:

P4, line 61: check Chegwidden and Carter ref
P4, line 64: Aspatwar et al., 2022 states only 8 classes of CA - they do not acknowledge the epsilon class.
P4, line 76: I don't think there is clear evidence this is a compensatory effect in microalgae and cyanobacteria. Rephrasing this sentence to more generally state that CCMs overcome the drawbacks of Rubisco would be more appropriate.
P4, line 79: "needed for photosynthesis" is a strange way to phrase this.
P4, line 85: a reference is needed for this statement - perhaps Sinetova et al., 2012, BBA?
P5, line 96: is -> was, reference for this also? Kimpel et al., (1983); Plant and Cell Phys., Coleman et al., (1984); Plant Phys./Fukuzawa et al., 1990; Nucl.Acid Res. ?
P6, line 120: LC abbreviation is not introduced
P6, line 126: it is unclear which condition the authors are referring to here
P6, line 143: for the reader it may be useful to explicitly state C9 has a cell wall.
P6, line 146: as above, it is essential that details of how this complementation were completed are included in the manuscript.
P7, line 154: Which media conditions (MOPS-P?, pH?) were used here. You later show that Ci affinity changes drastically in the mutants across pH changes so it is essential that the conditions are stated here.
P8, line 179: were only single isolates obtained in these experiments? If so, that's OK, but comparison of multiple colonies would be nice. The above-stated complementation of cah1-1 in these lines is essential though, particularly with only one isolate for each.
P8, lines 192-194: The author's say it themselves here, this is the major point that needs addressing.
P8, line 199: Do we actually have an idea of the membrane permeability of acetazolamide? In mammalian studies it is often cited as membrane-permeant (see Suparan et al., 2004; J Enz. Inhib. Med. Chem. etc.). See above comments.
P8, line 212: It is indeed surprising the reduce Ci-affinity has no effect on growth rate.
P9, line 233: This is important, and emphasises the importance of stating the conditions under which the RNA-seq experiments were completed.
P12, line 305: This is quite a big throwaway statement. If there are other compensatory mechanisms, why is the Ci-affinity

lower?

P6, lines 143-145: How do they know the LCR1 is knocked down?
P6, lines 145-147: There is currently no methodology
P6, line 146: there is no information on the complementation of LCR1, which promoter/terminators were used? - This could have a big impact on phenotype

P7, lines 158-160: Further analysis of this is needed. Currently the reader can't access the data for the lcr1 mutant and complemented lines.
P7, line 162: Is table 1 all the genes downregulated? Why were the other candidates ignored?

P8, line 186: These need to be complemented - this would also help address uncertainty around HLA3 contribution
P8, line 194: This is a major point that needs addressing.
P8, line 202: What is the mechanism for this? Free CA in the media replenishes CO2 in the equilibrium?

Reviewer #2 (Comments for the Author):

Title: Periplasmic carbonic anhydrase CAH1 contributes to high inorganic carbon affinity in Chlamydomonas reinhardtii. By Shimamura et al

This manuscript describes about the physiological role of an extracellular alfa-type carbonic anhydrase, CAH1 in the initial process of a biophysical CO2-concentrating mechanism (CCM) in the green alga, Chlamydomonas reinhardtii. The topic has a long/deep history from around 1980 and the role of CAH1 in the low-CO2-induced CCM has also been estimated by a number of investigations with relatively solid methods including knocking out of gene. As such mutation gave a marginal phenotype with trace difference of the photosynthetic Ci affinity and growth profiles relative to those of WT cells, the CAH1 role in the CCM has been somewhat controversial. The occurrences of other external CA, a very similar CAH2 and beta CAH8 also in some extents have obscured the CHA1 function. Nonetheless, a large body of through physiological data have demonstrated that CAH1 and/or CAH8 have a function to dehydrate external bicarbonate to help a diffusive CO2 entry across the plasma membrane. Later, this CO2 diffusion is hypothesized to be facilitated by LCI1, which initially is annotated as a bicarbonate transporter but then reannotated as a CO2 channel due to its structural features.
In this manuscript, the authors established KO mutants of Lcr1 (with greatly reduced level of lci1 and cah1), lci1, cah1, or lci1/cah1 from the same parental strain C9. The results well demonstrated that the lack of cah1 (but not lci1) significantly reduced the affinity of photosynthesis for Ci (5-8 times of the K0.5 value relative to WT), also ascribing the phenotype of the Lcr1 mutant to the absence of CAH1. This study is a through reconfirmation of the afore-mentioned CAH1 function but in this sense the main conclusions drawn in this manuscript are not strikingly new. Apart from CAH1, in the process of the new settings of this study, there are several new findings which potentially are important to consider molecular mechanisms of the external Ci acquisition. But it is unfortunate that such new findings are just only listed but are not extended in this study. Below I list my specific comments.

1. The authors claim one of significances of this experimental settings is the use of C9 strain for all mutagenesis. But the reason is described only briefly at L138-143 and 248-250. The confusion in the use of Chlamydomonas laboratory-keeping strains for many independent mutagenesis experiments is quite a niche topic that no one outside the field is familiar. The authors need to address what has been the problem in this issue in the history and what is solved in the present study.
2. L186 and anywhere related to this result: the silent phenotype of lci-1 mutants is new but this manuscript does not extend discussion on this point. There must be other candidate to help CO2 permeation and also possibility of LCI1 post-translational modifications.
3. Relating to above and Fig 4 model: It is interesting that this protein is potentially associated to HLA3, a bicarbonate transporter. What could be possible in Ci uptake regulations by this interaction? The HLA3 expression is also deeply related to the expression of LCIA, a bicarbonate transporter on the chloroplast envelope. Such multiple point controls of the HLA3 expression by other factors are an intriguing issue to be discussed in this manuscript.
4. Relating to above, looking at the Fig. 2B, only very trace amount of LCI1 and CAH1 proteins enables the normal accumulation of HLA3. In lcr1 mutant, are mRNA of these proteins must be completely missing or there is trace amount or derepressed? Is there any possibility of post transcriptional regulations in HLA expression by lci1, cah1, and lciA?
5. It is surprising that there is little difference in photosynthesis Ci affinity between lcr1 mutant and cah1 mutant. This implies that the contribution of HLA3 is little in total replenishment capacity against the cytosolic-Ci drawdown due to active uptake of Ci by chloroplast. Does this data agree with the KO mutant data of HLA3 in the previous work from (I think) the same group?
6. L215-217; 303-305: Please forward the discussion from the point of view of total Ci concentration in the culture medium under LC. Probably, total [Ci] in culture medium under atmospheric CO2 partial pressure could approach to the saturation Ci for photosynthesis when cell density is reasonably low in the culture medium.
7. L231-232: This is also a new point in this manuscript that may be discussed.
8. L276-281: Also a numerous physiological data are available from other algae. For instance, the relatively distant green algae Chlorella and other in 1990-2005.
9. L90: LCIB/C hexamer?

10. L96-97: first CA to be identified...: is it first in plants or algae? Please also quite reference here.
11. L177 and up: Is there any reason why Cre10.g426800 is only measured at pH 7.8?
12. L290: More specifically LCI1 is channel.
13. L293: It is helpful to explain that lcr1 mutant does not lose CAH1 and LCI1 but there is trace occurrence. From the western bolt data, it is difficult to convince it. So please quote appropriate reference or by supplemental material.
14. L314-316: This info needs to be written more in detail in Introduction.
15. L335: O2-evolving activity, not CO2-evolving.

EDITOR’S COMMENT

We have received reviews of your manuscript, which you may view at the end of this letter. On the basis of the advice received and my own evaluation, I believe that, pending revision, this article may ultimately be acceptable for publication in Plant Physiology. Both reviewers felt that the work was solid and the data clearly presented. However there was some concern over novelty, particularly from reviewer 2. Following consultation, both reviewers felt that the two experiments suggested by reviewer 1 could help to address these concerns. Please could you consider these and respond to the comments raised by both reviewers.

RESPONSE TO EDITOR’S COMMENT

We greatly appreciate your and the reviewers' time and insights on our manuscript. We are encouraged by the positive feedback on our work's solidity and data presentation. We have carefully addressed the concerns about novelty, particularly those raised by Reviewer #2. In response, we have conducted the two additional experiments suggested by Reviewer #1, which we believe significantly enhance our study's impact. We have thoroughly revised the text and figures to incorporate these new findings and address all reviewers' comments. The revised manuscript now offers clearer explanations and stronger conclusions. We are confident these changes have substantially improved our work and look forward to your evaluation. Thank you for your guidance and the opportunity to refine our research.

COMMENTS FROM THE REVIEWER #1

COMMENT 1-1

As the authors state, the *cah1-1* mutant displays reduced Ci affinity that can be rescued by the addition of extracellular carbonic anhydrase. However, the lack of HLA3 in the *cah1-1* mutant does not allow the authors to completely decouple the action of HLA3 and CAH1 at membrane, despite the CA inhibition experiments they present. Complementation of the *cah1-1* (and ideally the *lci1/cah1-1*) mutant and analysis of HLA3 presence and Ci affinity (and optionally growth phenotype) would allow a more convincing conclusion of this body of work. Secondly, much of the previous debate around CAH1's role has to do with conclusions from the use of CA inhibitors (acetazolamide). In this study, the authors leverage the same approach to conclude that CAH1 inhibition in the periplasm has the same effect as CAH1 knockout in the *cah1-1* line. In these experiments, the same acetazolamide treatment should be applied to the *cah1-1* line (and optionally the *cah1-1* supplemented with bovine CA experiment) to show that acetazolamide treatment does not further reduced Ci, which would be indicative of membrane permeability and the intracellular inhibition proposed by Williams and Turpin (1987).

RESPONE TO 1-1

Thank you for your valuable suggestion. We appreciate the opportunity to address these important points and strengthen our conclusions. As recommended, we have conducted additional experiments and revised our manuscript accordingly. We have performed the following new experiments:

- Generated a new *lci1*/*cah1*-1:*CAH1* complementation strain.
- Analyzed HLA3 presence and Ci affinity in the complemented strain.
- Applied AZA treatment to *cah1*-1 and *lci1*/*cah1*-1 mutants.
- Tested the effect of AZA on *cah1*-1 supplemented with bovine CA.

These new experiments have provided clearer insights into the specific roles of CAH1 and HLA3. Key findings include:

- The complemented *lci1*/*cah1*-1:*CAH1* strain showed restored CAH1 and HLA3

accumulation levels.

• AZA treatment of WT cells increased $K_{0.5}$ (Ci) to levels similar to *cah1*-1 and *lci1*/*cah1*-1 mutants.

• AZA treatment of *cah1*-1 and *lci1*/*cah1*-1 mutants did not further increase $K_{0.5}$ (Ci) values.

• The $K_{0.5}$ (Ci) values in *lci1*-1/*cah1*:CAH1 were similar to WT.

These results support our conclusion that the altered Ci-affinity in *cah1*-1 and *lci1*/*cah1*-1 is primarily due to the loss of CAH1 activity, rather than HLA3 deficiency. The lack of further effect from AZA treatment in *cah1*-1 mutants also addresses the concern about potential intracellular inhibition raised by Williams and Turpin (1987). We have revised the manuscript to include these new data and their interpretation, providing a more convincing conclusion about the critical role of periplasmic CAH1 in maintaining high-Ci affinity under CO_2-limiting conditions as follows:

(Line 211–230 in the revised manuscript)

“To further elucidate CAH1's specific contribution to Ci-affinity and separate its effects from those of HLA3, we investigated the response of cells treated with acetazolamide (AZA), a CA inhibitor with low membrane permeability, at pH 7.8. Additionally, we generated a new *lci1*/*cah1*-1:*CAH1* strain by introducing a gene fragment of *CAH1*, including its putative promoter, 5'-UTR and 3'-UTR, into *lci1*/*cah1*-1 for comparison. In *lci1*/*cah1*-1:*CAH1*, the accumulation levels of CAH1 and HLA3 were significantly increased compared to *lci1*/*cah1*-1(Fig. 2C). The addition of AZA to WT cells resulted in an increased $K_{0.5}$ (Ci) value, aligning with the levels observed in *cah1*-1 and *lci1*/*cah1*-1 mutants (Fig. 2D, E and Supplemental Table S2). Conversely, when *cah1*-1 and *lci1*/*cah1*-1 mutants were supplemented with bovine CA, their $K_{0.5}$ (Ci) value was reduced to WT levels, suggesting that exogenous CA activity can compensate for the loss of CAH1 function by replenishing CO_2 in the equilibrium. On the other hand, the addition of AZA to *cah1*-1 and *lci1*/*cah1*-1 cells did not cause a further significant increase in $K_{0.5}$ (Ci) values. Furthermore, $K_{0.5}$ (Ci) values in *lci1*-1/*cah1*:*CAH1* were similar to WT. In combination with previous studies showing a very minor contribution of HLA3 to the maintenance of Ci affinity at pH 7.8 (Yamano et al., 2015), our results demonstrate that the alteration in Ci-affinity observed in *cah1*-1 and *lci1*/*cah1*-1 is

primarily attributed to the loss of CAH1 activity. These results affirm the critical function of periplasmic CAH1 in CCM by maintaining high-Ci affinity under CO_2-limiting conditions."

COMMENT 1-2

Several other small additions are requested, but do not require additional experimentation:

- The complete RNA-seq data (including downregulated genes) for the *lcr1* and C-LCR1 lines should be included in Supplemental Dataset 1 to allow the reader to derive the values in Table 1.

RESPONSE TO 1-2

As suggested, we have expanded Supplemental Dataset 1. It now includes:

• Complete RNA-seq data for *lcr1*-1 and *lcr1*-1:*LCR1* (renamed from C-LCR1)

• Average TPM values for each gene

• Log_2FC and FDR values for comparisons: *lcr1*-1 vs WT and *lcr1*-1 vs *lcr1*-1:*LCR1*

These additions allow readers to verify the values in Table 1.

COMMENT 1-3

Details of how the complementation of *lcr1* was completed are missing. The promoter, UTRs and terminators used in this complementation are important to the complementation.

RESPONSE TO 1-3

As suggested, we have added details about the *lcr1* complementation in the Results section as follows:

(Line 153–156 in the revised manuscript)

"The *lcr1*-1:*LCR1* complemented strain was also created by reintroducing the *LCR1* gene fragment, including its putative promoter, 5'-UTR, and 3'-UTR, into *lcr1*-1. This restored the reduced CAH1 and LCI1 accumulation levels observed in *lcr1*-1 (Fig. 1)."

COMMENT 1-4

Details of the growth conditions (e.g. pH) for experiments are missing for the following experiments:

RNA-seq - which pH, which media?
Fig. 1 - which pH?
Fig. 2b - which pH?

RESPONSE TO 1-4

As suggested, we have added the information of medium and its pH in Results and Figure legends.

COMMENT 1-5

The oxygen evolution curves used to derive values in Figs. 2a, 2d and Supplemental Tables S1 and S2 should be provided in the supplementary data.

RESPONSE TO 1-5

As suggested, we have included the oxygen evolution curves as requested:

- Fig. 2E: Curves for data in Fig. 2A and 2D.
- Supplemental Figure S4: Curves for data in Supplemental Tables S1 and S2.

COMMENT 1-6

Additions to the methods are required in several places, as outlined below:
Photosynthetic O_2-evolving activity methods should at least cite a previous publication in which the same approach was used. There is an error in the title as well. The CRISPR reference Tsuji et al., 2022 doesn't exist - this should be 2023. Immunoblotting should cite the original reference (Wang et al., 2016, PNAS) which is cited in Shimamura et al., (2023).

RESPONSE TO 1-6

As suggested, we have made the following revisions to the Methods section:

- Added citation (Yamano et al., 2008 Plant Physiol.) for photosynthetic O_2-evolving activity measurement
- Corrected CRISPR reference to Tsuji et al., 2023
- Added original reference (Wang et al., 2016, PNAS) for immunoblotting
- Fixed title error

Thank you for bringing these issues to our attention.

COMMENT 1-7

For the RNA-seq experiments, were replicates used?

RESPONSE TO 1-7

We used two biological replicates for each condition in the RNA-seq experiments. We have revised the text in Materials and Methods as follows:

(Line 436–437 in the revised manuscript)

"In each condition, sequencing data were obtained from two biological replicates."

COMMENT 1-8

Although the CA inhibitor details are in the figure legend, it would be useful to have the details of these experiments in the methods section.

RESPONSE TO 1-8

As suggested, we have added CA inhibitor experiment details to the Methods section, including AZA and bovine CA concentrations.as follows:

(Line 412–415 in the revised manuscript)

"AZA adjusted to a concentration of 5 mM and dissolved in DMSO, was added to the measuring buffer at a 1% [v/v]. Bovine CA was added into the buffer, achieving a concentration of 2.0 μg mL^{-1}. For comparison, 1% DMSO was introduced to samples without AZA."

COMMENT 1-9

More discission as to why there is no phenotype are required. This is an important discrepancy between the Ci affinity and expected phenotype.

RESPONSE TO 1-9

As suggested, we have expanded the discussion on the lack of growth phenotype despite reduced Ci-affinity, suggesting future studies with varied CO_2 concentrations and pH conditions as follows:

(Line 370–377 in the revised manuscript)

"Despite the reduced Ci-affinity, the absence of significant growth rate differences among *lcr1*-1, *cah1*-1, and WT under CO_2-limiting conditions (Fig. 3A and B) suggests that the CO_2 concentrations used in our growth experiments were still sufficient to support normal growth in the mutants. This observation raises the possibility that the

growth conditions used in this study may not have been optimal for detecting the effects of CAH1 loss on growth rate. Future studies exploring a wider range of CO_2 concentrations and pH conditions are needed to fully elucidate the impact of CAH1 deficiency on growth under varying environmental conditions."

COMMENT 1-10

P4, line 61: check Chegwidden and Carter ref

P4, line 64: Aspatwar et al., 2022 states only 8 classes of CA - they do not acknowledge the epsilon class.

RESPONSE TO 1-10

We appreciate you pointing it out. As suggested, we have corrected the text to state that CA is classified into eight subclasses based on Aspatwar et al., 2022 as follows:

(Line 65–66 in the revised manuscript)

"CA is classified into eight subclasses based on the primary structure (Aspatwar et al., 2022)."

COMMENT 1-11

P4, line 76: I don't think there is clear evidence this is a compensatory effect in microalgae and cyanobacteria. Rephrasing this sentence to more generally state that CCMs overcome the drawbacks of Rubisco would be more appropriate.

RESPONSE TO 1-11

As suggested, we have rephrased the text to state that CCMs help overcome Rubisco's lower CO_2 affinity in aquatic organisms.as follows:

(Line 77−80 in the revised manuscript)

"In these aquatic organisms, the CCM helps overcome the disadvantage of Rubisco's lower CO_2 affinity by actively transporting HCO_3^- into the chloroplast stroma through membrane transporters and channels."

COMMENT 1-12

P4, line 79: "needed for photosynthesis" is a strange way to phrase this.

RESPONSE TO 1-12

We appreciate you pointing it out. As suggested, we have revised the text as follows:

(Line 80−81 in the revised manuscript)

"Once in proximity to Rubisco, HCO_3^- is converted to CO_2 by intracellular CA, effectively concentrating CO_2 (Raven et al., 2011)."

COMMENT 1-13

P4, line 85: a reference is needed for this statement - perhaps Sinetova et al., 2012, BBA?

RESPONSE TO 1-13

As suggested, we have added the reference in the text and the reference list (Sinetova et al., 2012).

COMMENT 1-14

P5, line 96: is -> was, reference for this also? Kimpel et al., (1983); Plant and Cell Phys., Coleman et al., (1984); Plant Phys./Fukuzawa et al., 1990; Nucl.Acid Res. ?

RESPONSE TO 1-14

As suggested, we have revised the text and added the Coleman et al., 1984 reference for CAH1 identification. as follows:

(Line 98−100 in the revised manuscript)

"CAH1, an α-type CA localized at the periplasmic space, was the first CA to be identified in *Chlamydomonas* (Coleman et al., 1984), but its importance in the CCM remains controversial."

COMMENT 1-15

P6, line 120: LC abbreviation is not introduced

RESPONSE TO 1-15

We appreciate you pointing it out. As suggested, we he introduced the LC (low-CO_2) abbreviation in the text as follows:

(Line 126−128 in the revised manuscript)

"We previously demonstrated through macroarray analysis, which is limited to a specific number of genes, that the *lcr1* mutant was unable to induce at least three low-

CO_2 (LC) inducible genes, namely *CAH1*, *LCI1*, and *LCI6* (Yoshioka et al., 2004)."

COMMENT 1-16

P6, line 126: it is unclear which condition the authors are referring to here

RESPONSE TO 1-16

We appreciate you pointing it out. As suggested, we have clarified that the *lcr1* mutant shows decreased Ci-affinity under CO_2-limiting conditions as follows:

(Line 133−134 in the revised manuscript)

"Although the *lcr1* mutant shows a decrease of Ci-affinity under CO_2-limiting conditions, the major contributor to this phenotype has not been determined yet."

COMMENT 1-17

P6, line 143: for the reader it may be useful to explicitly state C9 has a cell wall.

RESPONSE TO 1-17

As suggested, we have explicitly stated that the C9 strain has a cell wall as follows:

(Line 149−151 in the revised manuscript)

"To address this, we generated a new *lcr1* mutant, named *lcr1*-1 in this study, derived from the WT strain C9 with a cell wall."

COMMENT 1-18

P6, line 146: as above, it is essential that details of how this complementation were completed are included in the manuscript.

RESPONSE TO 1-18

We have added complementation details as mentioned in RESPONSE TO 1-3.

COMMENT 1-19

P7, line 154: Which media conditions (MOPS-P?, pH?) were used here. You later show that Ci affinity changes drastically in the mutants across pH changes so it is essential that the conditions are stated here.

RESPONSE TO1-19

We have added the information of medium and its pH as mentioned in RESPONSE TO

1-4.

COMMENT 1-20

P8, line 179: were only single isolates obtained in these experiments? If so, that's OK, but comparison of multiple colonies would be nice. The above-stated complementation of *cah1-1* in these lines is essential though, particularly with only one isolate for each. P8, lines 192-194: The author's say it themselves here, this is the major point that needs addressing.

RESPONSE TO 1-20

As mentioned in RESPONSE 1-1, we have performed additional experiments for *lci1*/*cah1*-1 complementation and revised the text accordingly.

COMMENT 1-21

P8, line 199: Do we actually have an idea of the membrane permeability of acetazolamide? In mammalian studies it is often cited as membrane-permeant (see Suparan et al., 2004; J Enz. Inhib. Med. Chem. etc.). See above comments.

RESPONSE TO 1-21

As mentioned in RESPONSE 1-1, we have performed additional experiments for *lci1*/*cah1*-1 complementation and revised the text accordingly.

COMMENT 1-22

P8, line 212: It is indeed surprising the reduce Ci-affinity has no effect on growth rate.

RESPONSE TO 1-22

We have revised the discussion on growth experiments as mentioned in RESPONSE TO 1-9.

COMMENT 1-23

P9, line 233: This is important, and emphasises the importance of stating the conditions under which the RNA-seq experiments were completed.

RESPONSE TO 1-23

As mentioned above in RESPONSE TO 1-4, we have added the information of the growth condition.

COMMENT 1-24

P12, line 305: This is quite a big throwaway statement. If there are other compensatory mechanisms, why is the Ci-affinity lower?

RESPONSE TO 1-24

We have omitted the mention of compensatory effects and revised the text on growth experiments in the Discussion section as mentioned above in RESPONSE TO 1-9.

COMMENT 1-25

P6, lines 143-145: How do they know the LCR1 is knocked down?

RESPONE TO 1-25

We have revised the text to show that *LCR1* expression was decreased in *lcr1*-1 and recovered in C-LCR1 as follows:

(Line 168–170 in the revised manuscript)

"Among them, under VLC conditions, the expression levels of 12 genes, including *LCR1*, *CAH1*, *LCI1*, and *LCI6*, were significantly decreased in *lcr1*-1 and recovered in C-*LCR1* (Table 1)."

COMMENT 1-26

P6, lines 145-147: There is currently no methodology

P6, line 146: there is no information on the complementation of LCR1, which promoter/terminators were used? - This could have a big impact on phenotype

RESPONSE TO 1-26

As mentioned above in RESPONSE TO 1-3, we have added information on LCR1 complementation, including promoter and terminator details.

COMMENT 1-27

P7, lines 158-160: Further analysis of this is needed. Currently the reader can't access the data for the lcr1 mutant and complemented lines.

RESPONSE TO 1-27

As mentioned above in RESPONSE TO 1-2, we have added more information to the Supplemental Dataset for reader access to *lcr1* mutant and complemented line data.

COMMENT 1-28

P7, line 162: Is table 1 all the genes downregulated? Why were the other candidates ignored?

RESPONSE TO 1-28

We chose to highlight *Cre10.g426800* due to its significant downregulation and potential functional relevance. While we acknowledge that other candidates may also be of interest, a comprehensive analysis of all downregulated genes falls beyond the scope of this current study, which focuses on the most prominently affected genes. In future studies, we plan to investigate these additional downregulated genes to provide a more comprehensive understanding of the *LCR1* mutation's effects. As suggested, we have revised the text to clarify why we focused on *Cre10.g426800* as follows:

(Line 170–173 in the revised manuscript)

"Among these genes, *Cre10.g426800*, encoding a protein of unknown function with a transferase domain, was particularly notable as its expression level decreased more than four-fold by LCR1 mutation both 0.3 h and 2 h after VLC induction (Table 1)."

COMMENT 1-29

P8, line 186: These need to be complemented - this would also help address uncertainty around HLA3 contribution

RESPONSE TO 1-29

As mentioned above in RESPONSE TO 1-1, we generated strains with restored CAH1 accumulation in *lci1*/*cah1*-1.

COMMENT 1-30

P8, line 194: This is a major point that needs addressing.

RESPONSE TO 1-30

As you rightly pointed out, whether the decrease in Ci affinity is caused by the deficiency of CAH1 is an important point. As mentioned above in RESPONSE TO 1-1, we have created complemented strains and added results to address the impact of CAH1 deficiency on Ci affinity.

COMMENT 1-31

P8, line 202: What is the mechanism for this? Free CA in the media replenishes CO_2 in the equilibrium?

RESPONSE TO 1-31

Yes. To address this point more clearly, we have revised the text to explain how exogenous CA activity compensates for the loss of CAH1 function by replenishing CO_2 in the equilibrium as follows:

(Line 219–222 in the revised manuscript)

"Conversely, when *cah1*-1 and *lci1*/*cah1*-1 mutants were supplemented with bovine CA, their $K_{0.5}$ (Ci) value was reduced to WT levels, suggesting that exogenous CA activity can compensate for the loss of CAH1 function to replenishes CO_2 in the equilibrium."

COMMENTS FROM THE REVIEWER #2

COMMENT 2-1

The authors claim one of significances of this experimental settings is the use of C9 strain for all mutagenesis. But the reason is described only briefly at L138-143 and 248-250. The confusion in the use of Chlamydomonas laboratory-keeping strains for many independent mutagenesis experiments is quite a niche topic that no one outside the field is familiar. The authors need to address what has been the problem in this issue in the history and what is solved in the present study.

RESPONSE TO 2-1

Thank you for your valuable comment. We appreciate the opportunity to clarify this important aspect of our experimental design. We understand that the significance of using the C9 strain for all mutagenesis might not be immediately apparent to readers outside the field of *Chlamydomonas* research. In response to your suggestion, we have expanded our explanation to provide more context and highlight the importance of this approach. We have added the following text to address the historical context and the problem solved in our study:

(Line 291−301 in the revised manuscript)

"Whole genome sequencing of various *Chlamydomonas* laboratory strains has revealed significant genetic diversity among these strains (Gallaher et al., 2015). Furthermore, we have previously reported results suggesting that WT strains can acquire characteristics during long-term subculturing (Tsuji et al., 2023). These findings underscore the potential for genetic drift and the accumulation of spontaneous mutations in laboratory strains over time. As demonstrated in our previous studies (Toyokawa et al, 2020; Tsuji et al., 2023), this study reaffirms the importance of using mutants generated from the same parental strain for accurate phenotypic analysis in *Chlamydomonas* reverse genetics. By using the C9 strain as the common background for all our mutants, we minimize the confounding effects of strain-specific genetic variations, ensuring more reliable and reproducible results."

COMMENT 2-2

L186 and anywhere related to this result: the silent phenotype of *lci-1* mutants is new

but this manuscript does not extend discussion on this point. There must be other candidate to help CO_2 permeation and also possibility of LCI1 post-translational modifications.

RESPONE TO 2-2

Thank you for your valuable comment. As suggested, we revised the text as follows:

(Line 342−353 in the revised manuscript)

“This unexpected result suggests a complex CO_2 uptake mechanism in *Chlamydomonas*. It is possible that unidentified CO_2 channels or transporters may compensate for the loss of LCI1. Additionally, functional redundancy in the Ci uptake system might allow other pathways to compensate for the deficiency of a single gene. Further analysis, such as creating multiple gene knockout mutants, could help elucidate the intricate nature of this Ci uptake system and the specific role of LCI1 within it. Additionally, post-translational modifications of LCI1 could play a crucial role in its function or regulation. Future studies investigating these aspects, including the identification of potential LCI1 interacting partners and analysis of its post-translational modifications, will be essential to fully understand the role of LCI1 in the CO_2 uptake mechanism of *Chlamydomonas*.”

COMMENT 2-3

Relating to above and Fig 4 model: It is interesting that this protein is potentially associated to HLA3, a bicarbonate transporter. What could be possible in Ci uptake regulations by this interaction? The HLA3 expression is also deeply related to the expression of LCIA, a bicarbonate transporter on the chloroplast envelope. Such multiple point controls of the HLA3 expression by other factors are an intriguing issue to be discussed in this manuscript. Relating to above, looking at the Fig. 2B, only very trace amount of LCI1 and CAH1 proteins enables the normal accumulation of HLA3. In *lcr1* mutant, are mRNA of these proteins must be completely missing or there is trace amount or derepressed? Is there any possibility of post transcriptional regulations in HLA expression by *lci1*, *cah1*, and *lciA*?

RESPONSE TO 2-3

Thank you for your valuable comment. Regarding your question about mRNA levels in

the *lcr1* mutant, we have included this information in Supplemental Dataset 1. Our data show that there are indeed trace amounts of *LCI1* and *CAH1* mRNA in the *lcr1* mutant, which could explain the normal accumulation of HLA3 in this mutant compared to *cah1*-1 and *lci1*-1. Your suggestion about the possibility of post-transcriptional regulation of HLA3 expression by *LCI1*, *CAH1*, and *LCIA* is also intriguing. While our current study does not directly address this aspect, it certainly warrants further investigation. In future studies, we plan to explore potential post-transcriptional regulatory mechanisms that might influence HLA3 expression and function. As suggested, we have revised the text and added Supplemental Data in the Discussion as follows:

(Line 354–366 in the revised manuscript)

"Interestingly, the accumulation level of HLA3 was reduced in *cah1*-1 and *lci1*-1 (Fig. 4). The complete loss of CAH1 and LCI1 may have caused this phenotype, as HLA3 accumulation was not altered in *lcr1*-1, which still expresses low levels of *CAH1* and *LCI1* (Fig. 1 and 2B, Supplemental Dataset 1). Since HLA3 and LCI1 interact and form a complex on the plasma membrane (Mackinder et al., 2017), it is possible that the formation of the HLA3-LCI1 complex was inhibited in *lci1*-1. Furthermore, the expression of *HLA3* is closely tied to that of LCIA, a HCO_3^- transporter located on the chloroplast envelope (Yamano et al., 2015). This multi-tiered control of HLA3 expression by various factors, including CAH1, LCI1 and LCIA, suggests a complex regulatory network governing Ci uptake. To further unravel the complexities of this regulatory network, future studies should investigate the physical interaction between CAH1 and HLA3, as well as the impact of LCIA, LCI1, and CAH1 deficiency on *HLA3* expression."

COMMENT 2-4

It is surprising that there is little difference in photosynthesis Ci affinity between *lcr1* mutant and *cah1* mutant. This implies that the contribution of HLA3 is little in total replenishment capacity against the cytosolic-Ci drawdown due to active uptake of Ci by chloroplast. Does this data agree with the KO mutant data of HLA3 in the previous work from (I think) the same group?

RESPONSE TO 2-4

Thank you for your insightful comment on the similar photosynthetic Ci affinity between *lcr1* and *cah1* mutants. You are correct that this observation suggests a less significant contribution of HLA3 to overall Ci uptake capacity than previously thought. Our data is consistent with our previous findings (Yamano et al., 2015 PNAS), which showed only a minor contribution of HLA3 to Ci affinity maintenance at pH 7.8. This consistency strengthens our current conclusions about the complex nature of the Ci uptake system in *Chlamydomonas*. In response to your comment, we have revised the text as follows:

(Line 225–228 in the revised manuscript)

"In combination with previous studies showing a very minor contribution of HLA3 to the maintenance of Ci affinity at pH 7.8 (Yamano et al., 2015), our results demonstrate that the alteration in Ci-affinity observed in *cah1*-1 and *lci1*/*cah1*-1 is primarily attributed to the loss of CAH1 activity."

COMMENT 2-5

L215-217; 303-305: Please forward the discussion from the point of view of total Ci concentration in the culture medium under LC. Probably, total [Ci] in culture medium under atmospheric CO_2 partial pressure could approach to the saturation Ci for photosynthesis when cell density is reasonably low in the culture medium.

RESPONSE TO 2-5

Thank you for your insightful comment regarding the total Ci concentration in the culture medium under low CO_2 (LC) conditions. Your point about the potential saturation of Ci for photosynthesis at low cell densities is well-taken. In light of this and other reviewers' comments, we have revised our discussion as follows:

(Line 370–377 in the revised manuscript)

"Despite the reduced Ci affinity, the absence of significant growth rate differences between *lcr1*-1, *cah1*-1, and WT under CO_2-limiting conditions (Fig. 3A and B) suggests that the CO_2 concentrations used in our growth experiments were still sufficient to support normal growth in the mutants. This raises the possibility that the growth conditions used in this study may not have been optimal for detecting the effects of CAH1 loss on growth rate. Future studies exploring a wider range of CO_2

concentrations and pH conditions are needed to fully elucidate the impact of CAH1 deficiency on growth under varying environmental conditions."

COMMENT 2-6

L231-232: This is also a new point in this manuscript that may be discussed.

RESPONSE TO 2-6

As suggested, we revised the text based on our previous work (Yamano et al. 2008 Plant Physiol.) as follows:

(Line 256–275 in the revised manuscript)

"Our finding that LCR1 does not regulate LHCSR3 under CO_2-limiting conditions builds upon our previous work (Yamano et al. 2008), which demonstrated the complex regulation of LHCSR3 (formerly known as *Li818r-1* and *Li818r-3*). In that study, we showed that LHCSR3 is induced by high light in a CCM1-independent manner, while under CO_2-limiting conditions, its expression is CCM1-dependent. The current results, showing that LCR1 (a downstream factor of CCM1) does not regulate *LHCSR3* under CO_2-limiting conditions, suggest a more intricate regulatory network. This implies that while CCM1 is involved in LHCSR3 regulation under CO_2-limiting conditions, it likely acts through factors other than LCR1.

To further elucidate the regulatory mechanism of LHCSR3 expression and the roles of CCM1 and LCR1 in this process, future studies should investigate the expression patterns of *LHCSR3* under various combinations of light intensity and CO_2 availability. Additionally, identifying transcription factors that mediate CCM1-dependent *LHCSR3* expression under CO_2-limiting conditions would be crucial. Exploring potential interactions between LCR1 and other transcription factors involved in *LHCSR3* regulation could also provide valuable insights. These investigations could reveal how *Chlamydomonas* fine-tunes its gene expression in response to complex environmental changes, particularly in the context of carbon concentration mechanisms and photoprotection."

COMMENT 2-7

L276-281: Also a numerous physiological data are available from other algae. For instance, the relatively distant green algae Chlorella and other in 1990-2005.

RESPONSE TO 2-7

As suggested, we added the following sentences to the discussion:

(Line 323–327 in the revised manuscript)

"Besides *Chlamydomonas*, the enhanced CO_2 uptake by periplasmic CA-mediated dehydration is supported in various algal species. In the relatively distant green alga *Chlorella*, physiological studies have provided evidence for the role of periplasmic CA (Matsuda et al., 2002). Furthermore, …"

COMMENT 2-8

9. L90: LCIB/C hexamer?

RESPONSE TO 2-8

As suggested, we have revised the text as follows:

(Line 91–92 in the revised manuscript)

"Additionally, the Low-CO_2 Inducible Protein B/C (LCIB/C) hexamer, a θ-type CA positioned around the pyrenoid, serves to reconvert CO_2 leaking from the pyrenoid into HCO_3^-, maintaining optimal Ci concentration for photosynthesis (Wang and Spalding, 2006; Yamano et al., 2010; Kasili et al., 2023)."

COMMENT 2-9

L96-97: first CA to be identified...: is it first in plants or algae? Please also quite reference here.

RESPONSE TO 2-9

As suggested, we have revised the text as follows:

(Line 98–100 in the revised manuscript)

"CAH1, an α-type CA localized at the periplasmic space, was the first CA to be identified in *Chlamydomonas* (Coleman et al., 1984), but its importance in the CCM remains controversial."

COMMENT 2-10

L177 and up: Is there any reason why Cre10.g426800 is only measured at pH 7.8?

RESPONSE TO 2-10

We appreciate your question about the pH conditions used for measuring *Cre10.g426800*. Our choice to measure at pH 7.8 was deliberate and based on several factors. CCM phenotypes in *Chlamydomonas* are most pronounced at slightly alkaline pH, with pH 7.8 being optimal for assessing CCM activity. We measured $K_{0.5}$ (Ci) values at this pH and found no significant increase in the mutants compared to WT, unlike in the *lcr1*-1 strain. Additionally, the complementation of *lcr1* with this gene restored the affinity to WT levels. These observations led us to conclude that the contribution of *Cre10.g426800* to CCM function is minimal. To reflect this reasoning, we have revised the text as follows:

(Line 186–192 in the revised manuscript)

"In these mutants, the $K_{0.5}$ (Ci) values, the Ci concentrations required for half-maximal O_2-evolving rate, were measured at pH 7.8, where CCM phenotypes are most pronounced. The mutants showed no significant increase in $K_{0.5}$ (Ci) compared to the WT, unlike in the *lcr1*-1 strain (Supplemental Table S1). Furthermore, complementation of *lcr1* with these genes restored Ci affinity to WT levels. These results suggest that these two genes were not important for maintaining high Ci-affinity under CCM-inducing conditions."

COMMENT 2-11

L290: More specifically LCI1 is channel.

RESPONSE TO 2-11

As suggested, we have revised the text as follows:

(Line 340–342 in the revised manuscript)

“Despite no significant reduction in Ci affinity in *lci1*-1 mutants (Fig. 2A and Supplemental Table S1), LCI1's cooperative role with other channels cannot be ruled out.”

COMMENT 2-12

L293: It is helpful to explain that *lcr1* mutant does not lose CAH1 and LCI1 but there is trace occurrence. From the western bolt data, it is difficult to convince it. So please quote appropriate reference or by supplemental material.

RESPONSE TO 2-12

As suggested, we have revised Supplemental Dataset 1 and revised the text as follows:

(Line 355–357 in the revised manuscript)

"The complete loss of CAH1 and LCI1 may have caused this phenotype, as HLA3 accumulation was not altered in *lcr1*-1, which still expresses low levels of *CAH1* and *LCI1*. (Fig. 1 and 2B, Supplemental Dataset 1)."

COMMENT 2-13

L314-316: This info needs to be written more in detail in Introduction.

RESPONSE TO 2-13

As suggested, we have revised the text in Introduction as follows:

(Line 103–112 in the revised manuscript)

"In addition to the abundant accumulation of CAH1 under CO_2-limiting conditions, inhibition of periplasmic CA by weakly permeable CA inhibitors, such as acetazolamide (AZA), has been shown to decrease Ci affinity (Moroney et al., 1985). While other CA isoforms, including the α-type CAH2 and the β-type CAH8, are also present in the periplasmic space, their expression levels under CO_2-limiting conditions are lower compared to that of *CAH1* (Moroney et al., 2011). These observations have led to the establishment of a well-known model in which periplasmic CA, particularly CAH1, facilitates diffusive CO_2 entry by maintaining a CO_2 gradient across the plasma membrane. This is achieved through the rapid equilibration of CO_2 with bulk HCO_3^- at the cell surface."

COMMENT 2-14

L335: O_2-evolving activity, not CO_2-evolving.

RESPONSE TO 2-14

We appreciate you pointing it out. As suggested, we have revised the text:

(Line 407 in the revised manuscript)

"Measurement of photosynthetic O_2-evolving activity"

30-Jul-2024
Dr. Takashi Yamano
Kyoto Daigaku
Kyoto 606-8502
Japan

MSID: PP2024-RA-00341R1
MS TITLE: Periplasmic carbonic anhydrase CAH1 contributes to high inorganic carbon affinity in Chlamydomonas reinhardtii

Dear Dr. Takashi Yamano:

I am pleased to inform you that your revised manuscript "Periplasmic carbonic anhydrase CAH1 contributes to high inorganic carbon affinity in Chlamydomonas reinhardtii" has been accepted for publication in Plant Physiology. Your manuscript will be checked for consistency with journal formatting and image standards by one of our Science Editors, who will contact you shortly. They may have a number of queries and suggestions for you. Please keep in mind that they work to increase the online visibility of your article and its attraction for readers from the widest of backgrounds. You will have the opportunity to review any revisions, and I hope you will work constructively with them to get the most out of your research. You will also have the opportunity to review all changes when you receive the author proofs for your article.

Please note the minor points to address by Reviewer #2 during the editorial process.

Once validated for publication, your article will be sent to a Science Editor for a final review of your manuscript and source files. After your final files are uploaded, your article will appear in the next available issue and the online Preview version of your article will be posted after your signed Copyright and Checklist forms and final source files are received.

Please be sure to include the following in your manuscript file:
Harvard style citations
List of all author contributions and funding information at the beginning of manuscript after the titles and author list.
List all supplemental materials associated with the article, with titles, at the end of the ms, before the references and acknowledgments section.
Complete Figure Legends for all figures appearing in the article listed before the Literature Cited.

Thank you and congratulations on your Plant Physiology paper!

Sincerely,
Editor, Plant Physiology

=====
FIRST AUTHOR PROFILES
=====

We recognize the hard work that goes into being First Author of a Plant Physiology paper by publishing a "First Author Feature" for each First Author alongside your paper.

If the first author(s) would like to submit information for a feature on Facebook and Twitter, fill out the form by clicking on the link below:

Plant Physiology First Author Feature Form: https://forms.office.com/r/ZBCGZfhm8F
If you cannot access the form through the link above, please contact Rachel at rbelsky@aspb.org.

To upload an image of yourself to accompany your profile, please put your photo in the correct journal folder at the link below. You must label your image: Author First Name Last Name Article Title.

First Author Profile Image Dropbox: https://www.dropbox.com/scl/fo/xz029p7qjr2jml2rp2kmq/h?dl=0&rlkey=6fkxcaqmxx35ap152b2osy1fz

=====
IMPORTANT REMINDER: PEER REVIEW REPORTS
=====

If you opted to publish a peer review report along with your article during the original submission process, it will be prepared by the editorial staff and publicly posted with your manuscript, inside the zip file that contains any other supplemental material. As a reminder, the peer review report is a public record of all comments from editors and reviewers, as well as your prior responses, as you received them in the decision letters for each draft of your manuscript. If you agreed to publish this report and have changed your mind, or are not sure if you selected this option, please contact the editorial office as soon as possible before signing the license agreement from our publisher.

-- Reviewer comments:
Reviewer #1 (Comments for the Author):

The authors have excellently addressed the only concerns I had with the manuscript. In my opinion the manuscript is suitable for publication. Thank you for the oppurtunity to review.

Reviewer #2 (Comments for the Author):

The revised manuscript well addressed both reviewers' concerns with a few additional experiments. Now the manuscript reads much clearer and points new findings in the Chlamydomonas CCM history. I have a few very minor concerns about wording and quote.

1. L80-81: Once in proximity to Rubisco, HCO3- is converted to CO2 by luminal CA within the pyrenoid tubules, effectively evolving CO2 (Raven et al., 2011).
2. L274: CCM originally stands for CO2 concentrating mechanism.
3. L327: Matsuda et al., 1999? 2002 is not on the reference.
4. L187-188: where HCO3- use capacity in the CCM phenotypes is most pronounced.
